# Genome-Wide Association Study Reveals Novel Genomic Regions Associated with 10 Grain Minerals in Synthetic Hexaploid Wheat

**DOI:** 10.3390/ijms19103237

**Published:** 2018-10-19

**Authors:** Madhav Bhatta, P. Stephen Baenziger, Brian M. Waters, Rachana Poudel, Vikas Belamkar, Jesse Poland, Alexey Morgounov

**Affiliations:** 1 Department of Agronomy and Horticulture, University of Nebraska, Lincoln, NE 68583-0915, USA; madhav.bhatta@huskers.unl.edu (M.B.); pbaenziger1@unl.edu (P.S.B.); bwaters2@unl.edu (B.M.W.); vikas.belamkar@unl.edu (V.B.); 2 Food Science and Technology Department, University of Nebraska, Lincoln, NE 68588-6205, USA; rpoudel2@huskers.unl.edu; 3 Wheat Genetics Resource Center, Department of Plant Pathology, Kansas State University, Manhattan, KS 66506-5502, USA; jpoland@ksu.edu; 4 International Maize and Wheat Improvement Center (CIMMYT), Emek, 06511 Ankara, Turkey

**Keywords:** *Triticum durum*, *Aegilops tauschii*, *Triticum aestivum*, marker-trait associations, genes, bread wheat, genetic biofortification, favorable alleles

## Abstract

Synthetic hexaploid wheat (SHW; *Triticum durum* L. × *Aegilops* *tauschii* Coss.) is a means of introducing novel genes/genomic regions into bread wheat (*T. aestivum* L.) and a potential genetic resource for improving grain mineral concentrations. We quantified 10 grain minerals (Ca, Cd, Cu, Co, Fe, Li, Mg, Mn, Ni, and Zn) using an inductively coupled mass spectrometer in 123 SHWs for a genome-wide association study (GWAS). A GWAS with 35,648 single nucleotide polymorphism (SNP) markers identified 92 marker-trait associations (MTAs), of which 60 were novel and 40 were within genes, and the genes underlying 20 MTAs had annotations suggesting a potential role in grain mineral concentration. Twenty-four MTAs on the D-genome were novel and showed the potential of *Ae. tauschii* for improving grain mineral concentrations such as Ca, Co, Cu, Li, Mg, Mn, and Ni. Interestingly, the large number of novel MTAs (36) identified on the AB genome of these SHWs indicated that there is a lot of variation yet to be explored and to be used in the A and B genome along with the D-genome. Regression analysis identified a positive correlation between a cumulative number of favorable alleles at MTA loci in a genotype and grain mineral concentration. Additionally, we identified multi-traits and stable MTAs and recommended 13 top 10% SHWs with a higher concentration of beneficial grain minerals (Cu, Fe, Mg, Mn, Ni, and Zn), a large number of favorable alleles compared to low ranking genotypes and checks that could be utilized in the breeding program for the genetic biofortification. This study will further enhance our understanding of the genetic architecture of grain minerals in wheat and related cereals.

## 1. Introduction

The global population is increasing rapidly and is expected to reach 9.8 billion in 2050 [1]. With the increase in global population, the demand for staple crops will continue to increase. Wheat (*Triticum aestivum* L.) is one of the most important staple crops, and it feeds more than one-third of the world’s population, providing carbohydrates, proteins, vitamins, antioxidants, fibers, and minerals [2]. In 2017/2018, wheat production was estimated at 756.8 million tons [3]. Despite the significant growth in wheat production, a large percentage of the population who rely on wheat as a staple crop suffer from deficiencies in minerals such as calcium (Ca), copper (Cu), iron (Fe), magnesium (Mg), and zinc (Zn) [4,5,6] because of the of low grain mineral concentrations [7]. Increased concentrations of essential minerals and decreased concentrations of toxic minerals such as cadmium (Cd) in wheat grain will have a significant impact on human health. One sustainable and cost-effective approach to increasing essential mineral concentration is through genetic biofortification, which requires identification of cultivars with useful genetic variability for grain minerals and understanding of the physiological and genetic architecture of these minerals in wheat [8].

Grain mineral concentration is dependent on several processes, including mineral absorption from the soil, uptake by the roots, translocation, assimilation, and remobilization to the seed [9]. The involvement of several processes for the accumulation of minerals in grain makes them complex traits, which are most likely controlled by many genes [8]. Quantitative trait loci (QTL) analysis or genome-wide association study (marker-trait associations; MTAs) approaches are widely used to dissect complex traits. In wheat, to date, 13 QTLs and 485 MTAs were identified for Ca [4,8,10], one QTL and 13 MTAs identified for Cd [11,12], 17 QTLs for Cu [8,10,13], 58 QTLs for Fe [5,8,10,13,14,15,16,17,18,19,20], three QTLs for Mg [8,10], 15 QTLs for manganese (Mn) [10,13], and 46 QTLs and 16 MTAs for Zn [5,8,10,13,14,15,16,17,18,19,20,21]. The identification of QTLs or MTAs for high concentrations of beneficial grain minerals such as Ca, Cu, cobalt (Co), Fe, lithium (Li), Mg, Mn, nickel (Ni), and Zn, and low Cd concentration will assist in genetic biofortification through marker-assisted selection and ultimately assist in ensuring nutritional security.

Improved wheat cultivars contain low concentrations of grain minerals [5] and have narrow genetic variation for grain minerals compared to wheat’s wild relatives [22]. Synthetic hexaploid wheat (SHW; *Triticum turgidum* L. × *Aegilops tauschii* Coss.) is being used as a means of introducing novel genes/genetic variation into bread wheat [23,24] and it is a potential source of high grain mineral concentrations [25]. Thus, we selected a panel of 123 synthetic hexaploid wheat genotypes to (i) explore the genetic variation of 10 grain minerals (Ca, Cd, Co, Cu, Fe, Li, Mg, Mn, Ni, and Zn) and grain protein concentration (GPC); (ii) identify marker–trait associations using a genome-wide association study (GWAS) and (iii) candidate genes containing nucleotide variants influencing grain minerals. This report is the first for Cu, Co, Fe, Li, Mg, Mn, and Ni in wheat. Results of this study will facilitate the selection of SHWs for use in wheat improvement programs and in enhancing the nutritive value through the integration of valuable grain mineral favorable alleles from SHWs to meet current and future dietary needs.

## 2. Results and Discussion

### 2.1. Phenotypic Variation for Grain Protein Content and Grain Minerals

Genotypic variability for GPC and grain minerals was assessed in 123 SHWs across two years (2016 and 2017) field studies in Turkey. The analysis of variance (ANOVA) combined over these years revealed a significant effect of genotype for all traits, whereas a significant genotype × year effect was observed for GPC, Ca, Cu, Mg, Mn, and Ni (Table 1). Non-significant genotype × year interactions for Cd, Co, Fe, Li, and Zn indicate the genetic stability of these traits across years. A wide range of genotypic variation for GPC and minerals was observed among the 123 SHWs (Table 1). A wide range of genetic variation observed for grain yield (GY) and thousand kernel weight (TKW) in these SHWs was described previously [26]. Variation for GPC ranged from 130 g∙kg^−1^ to 168 g∙kg^−1^ with an average of 151 g∙kg^−1^ in 2016 and from 116 g∙kg^−1^ to 169 g∙kg^−1^ with an average of 138 g∙kg^−1^ in 2017 (Table 1). Similarly, variation for grain Fe concentration combined over two years ranged from 17 to 65 mg∙kg^−1^ with an average of 39 mg∙kg^−1^ and for grain Zn concentration ranged from 10 to 39 mg∙kg^−1^ with an average of 23 mg∙kg^−1^. Some of these SHWs had higher grain Co, Cu, Fe, Li, and Mg concentrations, some had similar grain concentrations of Mn, Ni, and Zn, and some had lower grain Cd and Ca concentrations than previously reported in the Hard Winter Wheat Association Mapping Panel (HWWAMP) consisting of 299 diverse genotypes representing the USA Great Plains [12]. The lower concentration of grain Ca in the SHWs than in bread wheat cultivars has been reported previously [25]. A previous study had reported much higher grain Cd concentration (up to 0.6 mg∙kg^−1^) in winter wheat [12] than our study. The Cd concentration in the SHWs in this study was < 0.1 mg∙kg^−1^, which is below the regulatory toxic level of 0.2 mg∙kg^−1^. However, the low Cd concentration in SHWs may be reflective of low Cd concentration in the soil, and unless they are grown in a high Cd site, we cannot ascertain whether these lines will provide low-Cd alleles for breeding [12]. Additionally, a previous study on genetic variation for grain Fe, Mn, and Zn concentrations in SHWs reported between 25–30% higher grain mineral concentrations of Fe, Mn, and Zn than bread wheat cultivars and the higher grain mineral concentrations in SHWs were not only due to lower GYs, but also due to a higher nutrient uptake efficiency [25]. This result indicated that the SHWs are potential sources of high grain mineral concentrations and could be used for genetic biofortification of wheat.

Broad-sense heritability estimated across the two years was high (*H^2^* > 0.60) for GPC, Cu, Fe, Mg, Mn, and Zn concentrations; moderate (>0.40 and <0.60) for Ca and Ni concentrations, and low (<0.40) for Cd, Co, and Li concentrations (Table 1). Higher broad sense heritability indicated that the trait was largely governed by the genotypic effect. These results showed potential for the improvement of GPC, Cu, Fe, Mg, Mn, and Zn concentrations through phenotypic selection within SHWs. Similar heritability for these traits has been reported in previous studies [4,5,8,12,20].

### 2.2. Principal Component Analysis and Phenotypic Correlation

**Figure 1:**
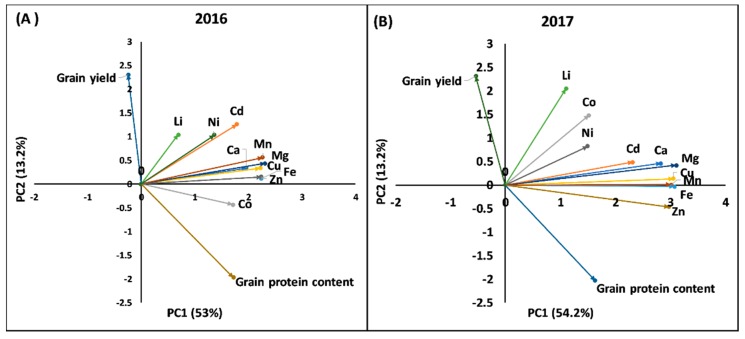
Factor analysis using the principal component method based on correlation matrices on grain yield, grain protein concentration, and 10 grain mineral concentrations in 123 synthetic hexaploid wheat lines grown in 2016 (**A**) and 2017 (**B**) in Konya, Turkey. PC1 = the first principal component analysis. PC2 = the second principal component analysis.

To understand the association among GY, GPC, and 10 mineral concentrations, a factor analysis using the principal component (PC) method was performed in each year (Figure 1). The first three PCs explained from 74.6% to 75.8% of the total variation in the data in 2016 and 2017, respectively. In 2016, the first PC explained 53.1% of the variation in the data and the variables included were Ca, Cd, Co, Cu, Fe, Mg, Mn, Ni, and Zn; the second PC explained 13.2% of the variation in data and variables included were GY and GPC; and the third PC explained 8.3% of the variation in the data and variable included was Li. Similarly, in 2017, the first PC explained 54.2% of the variation in the data and variables included were Ca, Cd, Cu, Fe, Mg, Mn, and Zn; the second PC explained 13.2% of the total variance and variables included were GY and GPC; and the third PC explained 8.4% of the total variance and the variables included were Co, Li, and Ni. Most of the grain minerals in both years were included in the first PC with positive loadings, implying that the first PC is a measure of overall mineral accumulations in the grain, which was similar to the conclusions of Guttieri et al. [12]. The second PC showed a negative correlation between GY and GPC. The association observed in the factor analysis was supported by the significant positive correlations (r) among most of the grain minerals and the negative correlation of GY and GPC (Table 2).

A significant negative correlation between GY and GPC was reported in previous studies [12,27] and the negative correlation was mainly due to the dilution effect. As expected, the present study also identified a significant negative correlation between GY and GPC (Table 2). Additionally, GY was positively correlated with TKW (*r* = 0.37, *p* < 0.0001 in 2016 and *r* = 0.35, *p* < 0.0001 in 2017), similar to previous studies [26,28]. However, GY was not correlated with grain minerals in this study whereas the significant negative correlation of GY with most of the grain minerals was observed after controlling for TKW (Table 2). This result indicated that TKW masked the true association of GY with minerals and controlling for the effect of TKW is important. Furthermore, canonical correlation analysis between GY and overall mineral concentration identified negative correlation (*r* = −0.37 in 2016 and *r* = 0.16 in 2017) between them. Similarly, several previous studies have identified a negative correlation of GY with grain minerals, including Fe [8,29] and Zn [8,10,12,29], which were reported to be associated with a dilution effect [12]. In the present study, GPC was significantly positively correlated with Ca, Cd, Cu, Fe, Mg, Mn, Ni, and Zn, however, the correlation was not very strong (0.51 ≥ r ≥ 0.19) (Table 2). Additionally, canonical correlation analysis between GPC and overall grain minerals identified a positive correlation (*r* = 0.44 in 2016 and *r* = 0.62 in 2017) between them. Many studies have shown a significant positive correlation of GPC with Fe and Zn concentrations [12,15,16,21], indicating that these traits might have a similar genetic basis and could be improved simultaneously [7]. Additionally, most of the grain minerals had highly significant positive correlations (*p* < 0.01) among each other. For instance, a strong correlation (*r* > 0.70, *p* < 0.0001) between Fe and Zn was observed, and they were also strongly correlated (*r* > 0.70, *p* < 0.0001) with other minerals such as Cu, Mg, Mn, and Zn. Positive correlations among grain minerals have been reported previously. For instance, many studies have shown the significant correlation between Fe and Zn concentrations in wheat [5,12,14,16,20,29]. However, other studies have shown no correlation between Fe and Zn [7,30], indicating the genotypic and environmental influence on the relationship between these traits. 

Cadmium is a toxic heavy metal that causes harm to human health. Reducing the grain Cd concentration is one of the important plant breeding objectives for creating healthier grains along with the enhancement of beneficial grain mineral concentrations [31]. The current study identified the significant positive correlation between grain Cd concentration with other minerals (Table 2). The previous study in HWWAMP (in this case, using 286 genotypes) also identified a significant positive correlation between grain Cd and Zn concentration, however, the correlation was not very strong (*r* < 0.49) [12]. However, independent genetic regulation of Cd and Zn has been reported [31], which may help explain this weak correlation. The current study identified a weak to moderate correlation (*r* < 0.70) of grain Cd concentration with other grain minerals, implying that enhancement of beneficial mineral concentration may be possible without further increasing grain Cd concentration.

### 2.3. Selection of Top-Ranking Genotypes

The 13 top 10% SHW lines were selected from two years of combined data that had higher amounts of GPC and beneficial grain mineral concentrations (Cu, Fe, Mg, Mn, Ni, and Zn) compared to checks and lower ranking genotypes and lower Cd concentration compared to lower ranking genotypes (Table S1). For instance, the Fe and Zn concentration in top ranking genotypes ranged from 49.5 to 56.0 mg∙kg^−1^ and 29 to 35 mg∙kg^−1^, respectively, whereas Cd concentration ranged from 0.07 to 0.08 mg∙kg^−1^. This result indicated that these genotypes could be used in the breeding program as parents with a goal of increasing beneficial grain minerals for addressing global mineral deficiencies while decreasing toxic compounds such as Cd.

### 2.4. Population Structure and Genome-Wide Association Study

Population structure analysis of the 123 SHWs was performed using 35,648 high quality genotyping-by-sequencing (GBS)-derived single nucleotide polymorphisms (SNPs) (minor allele frequency; MAF > 0.05 and missing data < 20%) that were well distributed across 21 chromosomes [26]. Our previous study on genetic diversity and population structure analysis of 101 SHWs identified a large amount of novel genetic variation that could be utilized in broadening the genetic diversity of bread wheat germplasms [23]. The population structure analysis identified that the 123 SHWs can be divided into three subgroups as described previously [26].

**Figure 2:**
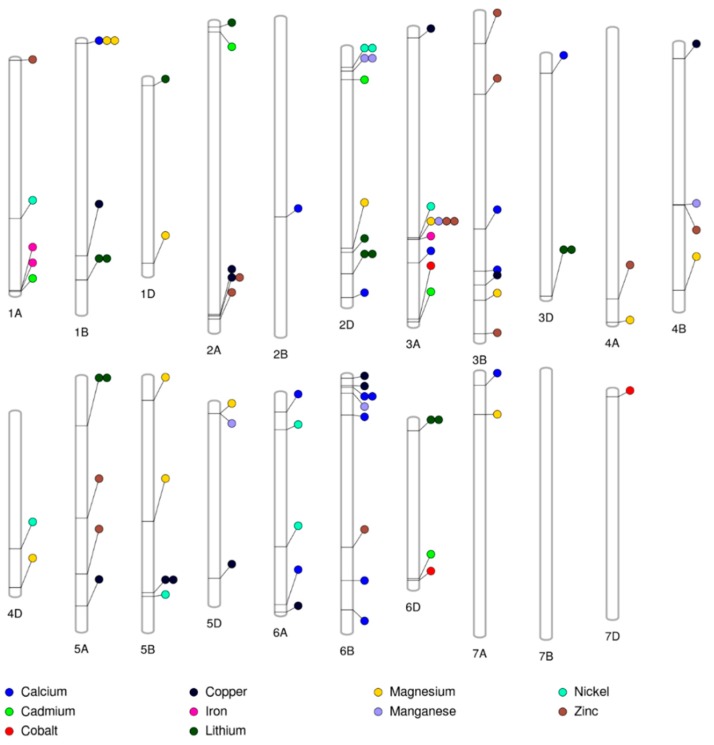
Significant marker–trait associations (MTAs) identified on each chromosome for 10 grain minerals from a genome-wide association study using 35,648 single nucleotide polymorphisms (SNPs) in 123 synthetic hexaploid wheat grown in 2016 and 2017 in Konya, Turkey.

The substantial genetic diversity in these SHWs and the dense SNP markers could be useful in identifying genetic factors underlying the variation for grain minerals using GWAS. A GWAS analysis performed using a multi-locus mixed linear model implemented in the FarmCPU algorithm with 35,648 GBS-derived SNPs for 10 grain minerals identified a total of 92 MTAs distributed across 20 chromosomes (Figure 2) with phenotypic variance explained (PVE) up to 25% (Table S2). Thirty-five MTAs were detected on the A genome, 32 MTAs on the B genome, and 25 MTAs on the D-genome of SHWs (Figure 2). The Manhattan and quantile–quantile (Q–Q) plots obtained from the GWAS were shown in Figure S1.

#### 2.4.1. Calcium

The 15 MTAs for Ca concentration were observed in 14 different genomic regions on chromosomes 1B, 2B, 2D, 3A, 3B, 3D, 6A, 6B, 7A (Figure 2) and the PVE by these MTAs ranged from 2.7% to 21.5% (Table S2), indicating a quantitative nature of inheritance for Ca concentration. Earlier studies have reported QTLs/MTAs for Ca on chromosomes 1A [8,10], 2A [10], 2D [10], 5A [4], 2B, 4A, 4B, 5B, 6A, and 7B [8] in wheat, indicating the involvement of these chromosomes in different mapping populations for Ca concentration. However, it is difficult to align our findings with earlier studies because of the employment of different marker systems (such as 90K SNP, short sequence repeat (SSR), and diversity arrays technology (DART)), the lack of precise location information in previous literature, or the utilization of a different version of the reference wheat genome other than the International Wheat Genome Sequencing Consortium (IWGSC) RefSeq v1.0 [26]. However, the associations identified on the same chromosomes as the previous study provided confidence in the reliability of these MTAs. The 11 MTAs identified in this study on chromosomes 1B, 3A, 3B, 3D, 6B, and 7A have not been reported and they are potentially novel MTAs controlling grain Ca concentration. Interestingly, no studies have identified a QTL in the D-genome.

#### 2.4.2. Cadmium

The five MTAs for Cd concentration were observed in five different genomic regions on chromosomes 1A, 2A, 2D, 3A, and 6D (Figure 2) with PVE ranging from 1.8% to 14.4% (Table S2). A previous study on QTL analysis in durum wheat identified a major QTL on chromosome 5B [11]. A GWAS was conducted using 286 winter wheat association mapping populations and identified 12 MTAs for Cd on chromosome 5A [31]. All five MTAs identified in this study are potentially novel MTAs controlling grain Cd concentration. Our study did not find the QTLs identified in the earlier studies; this may be due to the complexity of the trait and different genotypes used in this study. The identification of novel MTAs in the D-genome (2D and 6D) clearly represent variation from the *Ae. tauschii* and show the potential of SHW for its utilization in a marker-assisted breeding program upon validation in an independent genetic background.

#### 2.4.3. Cobalt, Lithium, and Nickel

The present study identified three MTAs on chromosomes 3A, 6D, and 7D for Co, 13 MTAs on chromosomes 1B, 1D, 2A, 2D, 3D, 5A, and 6D for Li, and eight MTAs on chromosomes 1A, 2D, 3A, 4D, 5B, and 6A for Ni (Figure 2 and Table S2). There is no previous report on QTL or GWAS analysis for Co, Li, and Ni in wheat. Therefore, all the MTAs identified for Co, Li, and Ni are potentially novel MTAs responsible for Co, Li, and Ni concentrations. Interestingly, our study identified several MTAs on the D-genome for Co, Li, and Ni, which showed the utility of SHWs for the improvement of these traits.

#### 2.4.4. Copper

A total of 13 MTAs for Cu were identified on chromosomes 1B, 2A, 3A, 3B, 4B, 5A, 5B, 5D, 6A, and 6B (Figure 2) with PVE ranging from 1.2% to 17.1% (Table S2). Earlier studies have identified one QTL for Cu concentration on chromosome 5A in diploid wheat (*T. monococum*) [13], 10 QTLs on chromosomes 1A, 2A, 3B, 4A, 4B, 5A, 6A, 6B, 7A, and 7B in tetraploid wheat [8], and six QTLs on chromosomes 2A, 4A, 4D, 5A, 6A, and 7B in hexaploid wheat [10]. The five MTAs identified in this study on chromosomes 1B, 3A, 5B, and 5D have not been reported and they are potentially novel MTAs controlling grain Cu concentration.

#### 2.4.5. Iron

A total of three MTAs for Fe concentration were identified on chromosomes 1A and 3A (Figure 2) with PVE ranging from 11.2% to 13.2% (Table S2). Earlier studies have identified 58 QTLs distributed on 16 chromosomes (1A, 1B, 2A, 2B, 2D, 3A, 3B, 3D, 4B, 4D, 5A, 5B, 6A, 6B, 6D, 7A, 7B, and 7D) [5,8,10,13,14,15,16,17,18,19,20].

#### 2.4.6. Magnesium

A total of 13 MTAs for Mg concentration were identified on chromosomes 1B, 1D, 2D, 3A, 3B, 4A, 4B, 4D, 5B, 5D, and 7A (Figure 2) with PVE ranging from 1.4% to 14.6% (Table S2). Earlier studies have identified eight QTLs for Mg concentration on chromosomes 1B, 2A, 3A, 5B, 6A, 6B, 7A, and 7B in tetraploid wheat [8] and three QTLs on chromosomes 4A, 5A, and 6A in hexaploid wheat [10]. The six MTAs identified in this study on chromosomes 1D, 2D, 3B, 4B, 4D, and 5D have not been reported and they are potentially novel MTAs controlling grain Mg concentration.

#### 2.4.7. Manganese

A total of six MTAs for Mn concentration were identified on chromosomes 2D, 3A, 4B, 5D, and 6B (Figure 2) with PVE ranging from 4.4% to 14.3% (Table S2). Earlier studies have identified one QTL on chromosome 5A in *T. monoccocum* [13], two QTLs for Mn concentration on chromosomes 2B and 7B in tetraploid wheat [8] and four QTLs on chromosomes 1A, 2B, 3B in hexaploid wheat [10]. All the six MTAs identified in this study on chromosomes 2D, 3A, 4B, 5D, and 6B have not been reported and they are potentially novel MTAs controlling grain Mn concentration.

#### 2.4.8. Zinc

A total of 13 MTAs for Zn concentration were identified on chromosomes 1A, 2A, 3A, 3B, 4A, 4B, 5A, and 6B (Figure 2) with PVE ranging from 1.8% to 14.1% (Table S2). Earlier studies have identified 46 QTLs on 15 chromosomes 1A, 1B, 1D, 2A, 2B, 3A, 3D, 4A, 4B, 4D, 5A, 6A, 6B, 7A, and 7B for Zn concentration [5,8,10,13,14,15,16,17,18,19,20,21]. Additionally, a previous GWAS for Zn concentration identified 13 MTAs on chromosomes 1B, 3A, and 4B [31]. Three MTAs identified in this study on chromosome 3B have not been reported and they are potentially novel MTAs controlling grain Zn concentration.

### 2.5. Relationship Between Grain Mineral Concentrations and Number of Favorable Alleles

**Figure 3:**
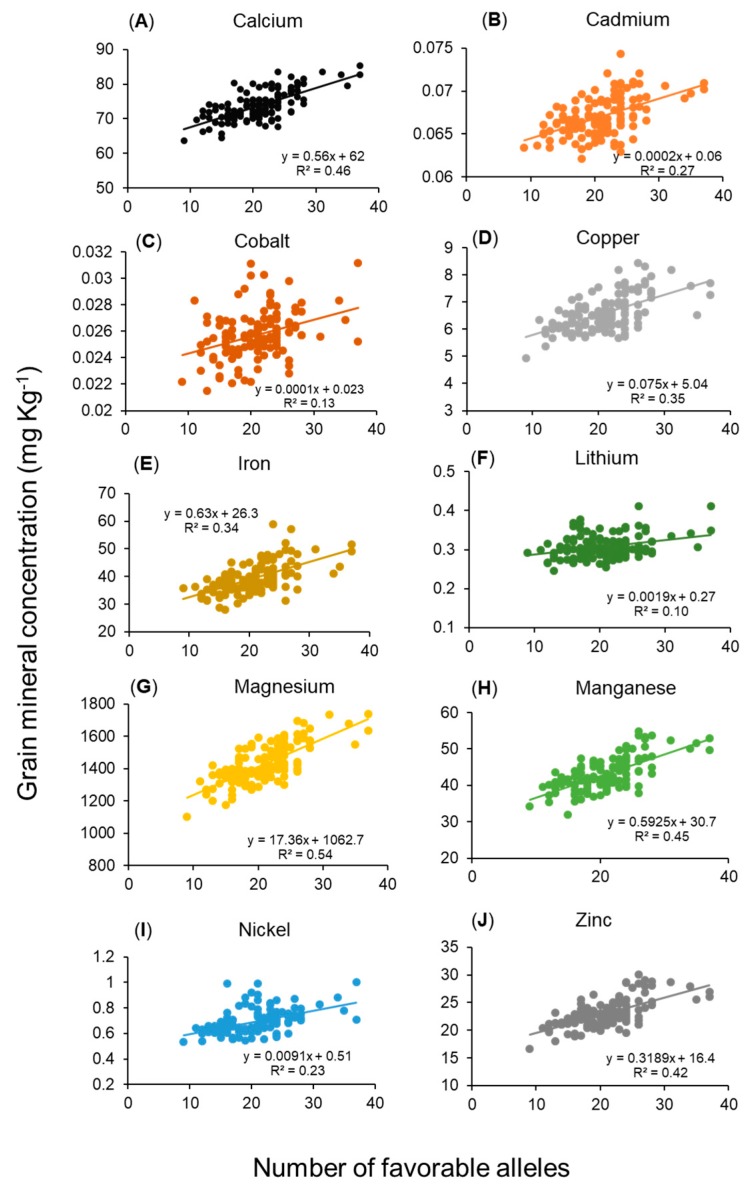
Regression analysis between the total number of favorable alleles per genotype and best linear unbiased predictor values of grain mineral concentrations obtained from two years (2016 and 2017) of experiments conducted in Konya, Turkey. The number of favorable alleles is the total number of alleles present in a genotype that increases the grain concentration of beneficial minerals such as calcium (**A**), cobalt (**C**), copper (**D**), iron (**E**), lithium (**F**), magnesium (**G**), manganese (**H**), nickel (**I**), and zinc (**J**), while decreasing grain cadmium (**B**) concentration.

The number of favorable alleles in a genotype is the cumulative number of alleles from MTAs that increase the concentration of beneficial minerals while decreasing the Cd concentration. A linear relationship between grain mineral concentration and the number of favorable alleles per genotype was observed (Figure 3), implying that the addition of every favorable allele in a genotype contributed to increasing beneficial grain mineral concentrations while decreasing grain Cd concentration. The number of favorable alleles within 123 SHWs ranged from 9 to 37 alleles (Table S3) and variance explained (*R^2^*) by favorable alleles on grain minerals ranged from 10% to 53% (Figure 3). The top-ranking 13 genotypes have a high number of favorable alleles, ranging from 23 to 27 alleles (Table S1). This result suggested that pyramiding these favorable alleles can enhance the grain mineral concentration and be used in a breeding program for genetic biofortification.

### 2.6. Multi-Trait and Stable Marker-Trait Associations

The present study identified common regions associated with multiple traits on chromosomes 1B, 2A, 3A, and 5B (Figure 2 and Table S2). For instance, the MTA for Ca and Mg was identified on chromosome 1B at 6,867,825 bp, Cu and Zn on chromosomes 2A at 742,969,119 bp, Cu and Mg on chromosome 5B at 607,870,649 bp, and Mg, Mn, and Zn on chromosome 3A at 534,469,328 bp. The co-locations of MTAs for Ca, Cu, Mg, Mn, and Zn indicated the same genomic region controlling these traits, which was also supported by highly significant strong positive correlations among those minerals (Table 2). These results suggested that the relationship among these traits was at the molecular level, indicating a common genetic basis for these traits which could be improved simultaneously. The QTL co-localization for some of the minerals have previously been reported. The co-localization of grain Zn QTLs with grain Fe QTLs in tetraploid wheat [8] and hexaploid wheat [5,16,20,21], and QTLs for Mn co-located with Fe concentration in tetraploid wheat [8] have been observed. Co-localization may have occurred either by pleiotropy of the same gene involved in controlling mineral concentrations of different elements, or by the presence of different linked genes in the same regions controlling mineral concentrations of different elements independently. Although Cd was significantly associated with grain minerals, we did not find co-localization of grain Cd MTAs with the MTAs of other grain minerals, indicating that the grain Cd concentration may be governed by a different genetic mechanism, as described previously [31].

In this study, we identified a stable MTA for Ca on chromosome 6B (32,333,184 bp), Cu on 5B (607,870,649 bp), Mg on 1B (867,825 bp), Mn on 2D (58,740,285 bp), Ni on 2D (48,611,294 bp), Zn on 3A (534,469,328 bp), and five stable MTAs for Li on chromosomes 1B (606,491,241 bp), 2D (572,031,650 bp), 3D (610,567,350 bp), 5A (135,164,381 bp), and 6D (30,744,756 bp) (Table S2). The stable MTAs identified could be used for the genetic improvement of these traits.

### 2.7. Genes Underlying Marker-Trait Associations

The MTAs that were identified were searched against the IWGSC RefSeq v1.0 annotation to identify genes underlying the various MTAs identified in this study. Identification of underlying genes with annotations matching the trait function would provide further confidence for these MTAs. The 40 MTAs (Ca: 8 MTAs; Cd: 1 MTA; Co: 2 MTAs; Cu: 4 MTAs; Fe: 3 MTAs; Li: 4 MTAs; Mg: 5 MTAs; Mn: 3 MTAs; Ni: 3 MTAs; and Zn: 7 MTAs) for 10 grain minerals were found within genes distributed on chromosomes 1A, 1B, 2A, 2D, 3A, 3B, 4A, 4B, 4D, 5A, 6A, 6B, 6D, and 7A (Table S4). Of these, 28 MTAs were present in 19 genes whose annotations indicate they are associated with grain minerals (Table 3 and Table S4). For instance, MTAs for Fe were located in genes related to Fe concentration such as 2-oxoglutarate (2OG), Fe(II)-dependent oxygenase superfamily protein [32,33], ATP synthase gamma chain [34], F-box family protein domain [33], GDSL esterase/lipase [35], leucine-rich receptor-like protein kinase [36,37], Myb transcription factor [36,37], Na-translocating NADH–quinone reductase subunit A [38], no apical meristem (NAM) protein [39], protein DETOXIFICATION [37], ROP guanine nucleotide exchange factor 10 [33], and universal stress protein family [37]. Additional examples are provided in Table 3. This result provides further evidence for these MTAs and indicates that these genes could be important for grain minerals in wheat, however, functional characterization studies are needed to validate the function of these genes.

Furthermore, we identified several MTAs for the same or multiple traits located within genes that had the same gene annotations (Table 3 and Table S2). For instance, some of the MTAs for the same traits such as Mg on chromosomes 3B and 4D, and Zn on chromosomes 3B and 6B were within genes that were both annotated as F-box family protein domain. Similarly, some of the MTAs for multiple traits such as Ca (1 MTA) on chromosome 6B, Li (1 MTA) on 2D, Mg (1 MTA) on 4A, and Zn (1 MTA) on 3B were within genes annotated as leucine-rich repeat receptor-like protein kinase (Table 3), indicating that these genes may be important for improving multiple traits. Multiple MTAs for different traits within genes having the same gene annotation was also reported in our previous study on drought stress related-traits [26].

## 3. Materials and Methods

### 3.1. Plant Materials and Experimental Design

The detail of the experimental materials and design were described previously [26]. In brief, a diversity panel of 123 SHWs originating from the International Maize and Wheat Improvement Center (CIMMYT), Mexico and Kyoto University, Japan were used (Table S3). Grain samples from each plot were obtained from field trials conducted in 2016 and 2017 growing seasons at the research farm located at the Bahri Dagdas International Agricultural Research Institute in Konya, Turkey (37°51′15.894′′ N, 32°34′3.936′′ E; elevation = 1021 m). The mean monthly temperature in both growing seasons was similar [26], however, the total rainfall in the 2017 growing season (243 mm) was slightly higher than that observed in the 2016 growing season (222 mm) [26]. However, rainfall in both growing seasons was below the 25-year average (435 mm), suggesting the presence of drought-stressed environmental conditions [26]. The soil texture was clayey loam, with a mean pH of 7.7 in 2016 and 8.2 in 2017 (Table S5). Details on soil analysis are provided in Table S5. The experimental design in the 2016 growing season was an augmented design with replicated checks (“Gerek” and “Karahan”) and modified alpha lattice design with replicated checks (Gerek and Karahan) and two replications in 2017 as described previously [26].

### 3.2. Grain Yield, Thousand Kernel Weight, Grain Protein Concentration, and Grain Mineral Analysis

Grain yield, TKW, and GPC were measured using previously reported protocols [26,27,28]. Whole grain mineral analysis was performed as described previously [12]. In brief, approximately 2 g of oven dried grains were digested with concentrated nitric acid (Optima, Fisher Chemical, Thermo Fisher Scientific Inc., Waltham, MA, USA) and hydrogen peroxide (30% H_2_O_2_, Fisher BioReagents, Thermo Fisher Scientific Inc., Waltham, MA, USA). Each digestion set of 50 samples included a reagent blank and 0.25 g of standard reference flour (standard reference material 1567a, National Bureau of Standards, MD). Grain mineral concentrations were determined in duplicate by inductively-coupled plasma mass spectrometry (ICP-MS; Agilent 7500cx, Agilent Technologies Inc., Santa Clara, CA, USA) with Ar carrier and a He collision cell at the University of Nebraska Redox Biology Center, Proteomics and Metabolomics Core. Mineral concentrations for Ca, Cd, Co, Cu, Fe, Li, Mg, Mn, Ni, and Zn were averaged over the duplicates and a reagent blank was subtracted. Mineral concentrations expressed as mg∙kg^−1^ (dry weight basis) were used for further analysis [31].

### 3.3. Phenotypic Data Analysis

Combined over two years, individual year analyses of variance (ANOVA) were computed using a mixed linear model using PROC MIXED in SAS 9.4 [47]. This was performed to estimate the best linear unbiased predictors (BLUPs) and to determine whether significant variations exist among the genotype, year, and their interactions. The details of the mixed linear model used for the analysis was described previously [26]. In brief, for the combined ANOVA, year and check were assumed as fixed effects whereas genotype, genotype x year interaction, replication nested within a year, and incomplete block nested within replications were assumed as random effects. For augmented design in 2016, ANOVA was calculated by assuming check as a fixed effect whereas genotype and incomplete block were assumed as random effects. Incomplete blocks nested within replication, checks fitted into new variable (new variable: check was coded as 0 and entry was coded as 1, where genotype was taken as a new variable x entry), and replications were used to correct for spatial variation in the data. For modified alpha (α) lattice design in 2017, ANOVA was calculated by assuming check as a fixed effect and genotype, replication, and incomplete block nested within replication as random effects. Broad-sense heritability was calculated based on an entry mean basis using the following formula:

(1) $$\;H^{2}=\frac{\sigma^{2}g}{\sigma^{2}g+\frac{\sigma^{2}\mathrm{yr}}{n}+\frac{\sigma^{2}\mathrm{gxyr}}{\mathrm{nr}}}\;$$

where, σ^2^_g_, σ^2^_yr_, and σ^2^_gxyr_ are the variance components for genotype, year, and genotype x year, respectively, and n and r are the number of years and replications, respectively.

The phenotypic correlation was computed using PROC CORR in SAS using BLUPs of each trait. To understand the association among grain minerals, GPC, and GY, a factor analysis using the principal component (PC) method and varimax rotation was performed on the correlation matrix in each year using the factoextra package in R software [48]. Furthermore, canonical correlation was performed between GY/GPC and mineral concentrations to determine the relationship between GY/GPC and overall mineral concentration.

### 3.4. Genotyping and SNP Discovery

Genotyping, SNP discovery, and SNP filtering procedures were described previously [23]. Briefly, DNA was extracted from fresh young leaves (approximately 14 days after sowing) using BioSprint 96 Plant Kits (Qiagen, Hombrechtikon, Switzerland). Genotyping was performed using a genotyping-by-sequencing approach [49]. SNP discovery was performed using TASSEL v. 5.2.40 GBS v2 Pipeline [50] with a physical alignment to the Chinese spring genome sequence (RefSeq v1.0) provided by the IWGSC [51]. The identified SNPs were filtered for MAF (>5%) and missing data (<20%) [23,52]. The GBS-derived SNP markers used in this study were provided previously [26].

### 3.5. Population Structure and Genome-Wide Association Analysis

The population structure analysis was described in our previous study [23]. In brief, the population structure of 123 genotypes was assessed using STRUCTURE v 2.3.4 [53] and the unweighted pair group method with arithmetic mean using TASSEL [54].

A GWAS was performed separately for each year (BLUPs from 2016 (BLUP16), BLUPs from 2017 (BLUP17), and BLUPs combined over years (CBLUP)) (Table S6) to identify MTAs for grain minerals using FarmCPU (fixed and random model circulating probability unification) with population structure (Q_1–3_) as a fixed effect (covariate) and FarmCPU calculated kinship as a random effect [55] implemented in the MVP R software package (available online: https://github.com/XiaoleiLiuBio/MVP). The identified MTAs were tested against a Bonferroni correction at a 5% level of significance with a *p* = 1.4026 × 10^−6^ (−log_10_*p* = 5.85) for multiple testing correction. Regression analysis was performed between the cumulative number of favorable alleles in a genotype and the BLUPs of each trait. Functional annotations of genes were retrieved using the IWGSC RefSeq v1.0 annotations provided for Chinese spring [51]. The impact of nucleotide variants on predicted genes or proteins was investigated using SnpEff software (available online: http://snpeff.sourceforge.net/).

## 4. Conclusions

The SHWs under study are a valuable resource for the genetic improvement of wheat because they were reported to have large amounts of novel genetic diversity (including D-genome diversity [23]), are resistant to multiple stresses [24,26], and showed a weak correlation of GY with GPC and most of the minerals, indicating improvement of grain minerals and GPC without sacrificing yield could be possible. Further, the strong positive correlations observed among the most grain minerals suggested the simultaneous improvement of grain minerals could be possible. The top ranking 13 genotypes with higher concentrations of useful grain minerals and lower concentrations of Cd identified in this study have a large number of favorable alleles and these SHWs could be used as a donor parent in a wheat breeding program for genetic biofortification.

A GWAS identified 92 MTAs, of which 60 MTAs were novel (15 MTAs on the A genome, 21 MTAs on the B genome and 24 MTAs on the D genome). The large number of novel MTAs (36) identified in the AB genome of these SHWs indicated that there is a lot of variation yet to be explored and to be used in the A and B genome. Several MTAs identified in this study were within genes with potential roles in improving grain mineral concentrations based on information for their annotations in the literature, which provided further evidence for the reliability and usefulness of the MTAs identified. However, further investigation on identified genomic regions could significantly assist in genetic biofortification program. Interestingly, this study identified several MTAs for grain minerals located in genes on different chromosomes that had the same gene annotation, suggesting that the same gene family may play a major role in affecting different grain mineral concentrations in SHWs. This study identified multi-trait (Ca and Mg; Cu and Mg; Mg and Cu; Mg, Mn, and Zn) MTAs on chromosomes 1B, 2A, 3A, and 5B which suggested a common genetic basis of these traits, showing the possibility of simultaneous improvement of these traits. Additionally, we identified several stable MTAs for Ca, Cu, Li, Mg, Mn, Ni, and Zn that could be used for the genetic improvement of grain minerals. In summary, a wide range of useful genetic variations for grain minerals and identification of several stable, co-localized, multi-trait, and novel genomic regions (especially on the D-genome) demonstrate the potential of SHWs in its utilization in the wheat breeding program for the genetic biofortification and this study also provided information towards further understanding the genetic complexity of grain mineral accumulation in wheat.

## Figures and Tables

**Table 1:** Analysis of variance and phenotypic variation for 10 grain minerals, grain protein concentration, and grain yield with minimum (min), maximum (max), fold change (max/min), mean, and broad sense heritability (*H^2^*) values of 123 synthetic hexaploid wheat (SHW) grown in 2016 and 2017 in Konya, Turkey.

Trait	2016				2017				Trials Combined			
	Min	Max	Fold	Mean	Min	Max	Fold	Mean	Year (Yr)	Genotype (G)	G x Yr	*H^2^*
Ca (mg∙kg^−1^)	47.5	167.2	3.5	103.1	21.6	84.5	3.9	44.3	*	**	***	0.41
Cd (mg∙kg^−^^1^)	0.03	0.1	3.44	0.07	0.02	0.13	7.68	0.07	NS	*	NS	0.28
Co (mg∙kg^−1^)	0.01	0.06	6.53	0.03	0.01	0.04	6.86	0.02	***	*	NS	0.33
Cu (mg∙kg^−1^)	2.8	11.4	4.1	7.5	2.9	8.9	3.1	5.7	NS	***	***	0.63
Fe (mg∙kg^−1^)	17.7	61.8	3.5	40.2	15.4	67.7	4.4	38.5	NS	***	NS	0.78
Li (mg∙kg^−1^)	0.04	0.23	6.43	0.09	0.13	1.07	8.43	0.52	***	*	NS	0.35
Mg (mg∙kg^−1^)	617	2097	3	1391	659	2131	3	1458	NS	***	**	0.62
Mn (mg∙kg^−1^)	20.3	66.2	3.3	41.2	21.5	69.8	3.2	44.9	NS	***	**	0.67
Ni (mg∙kg^−1^)	0.21	2.22	10.81	0.91	0.13	1.16	8.75	0.48	NS	***	***	0.52
Zn (mg∙kg^−1^)	8.8	38.1	4.3	23.1	11.1	39.6	3.6	23	NS	***	NS	0.65
Grain protein (g∙kg^−1^)	129.8	167.6	1.3	151.2	116.4	168.9	1.5	137.8	***	***	**	0.68
Grain yield (g∙m^−2^)	54.3	530	9.8	259	194.7	479.5	2.5	290.1	NS	*	*	0.44

*, **, and *** = significant at the 0.05, 0.01, and 0.001 probability level, respectively; NS = non-significant at the 0.05 probability level.

**Table 2:** Pearson’s correlation coefficients of 10 grain minerals, grain protein content (GPC), grain yield (GY), and GY controlling for thousand kernel weight (GY_pTKW_) in 123 synthetic hexaploid wheat grown in 2016 (upper triangle) and 2017 (lower triangle) in Konya, Turkey.

Trait	Ca	Cd	Co	Cu	Fe	Li	Mg	Mn	Ni	Zn	GPC	GY	GY_pTKW_
Ca	1	0.63 ***	0.42 ***	0.64 ***	0.58 ***	0.31 ***	0.80 ***	0.79 ***	0.44 ***	0.60 ***	0.36 ***	−0.01	−0.11
Cd	0.64 ***	1	0.32 ***	0.68 ***	0.61 ***	0.38 ***	0.65 ***	0.67 ***	0.58 ***	0.63 ***	0.22 *	−0.03	−0.40 ***
Co	0.37 ***	0.34 ***	1	0.49 ***	0.63 ***	0.19 *	0.49 ***	0.49 ***	0.54 ***	0.53 ***	−0.05	0.13	0.01
Cu	0.82 ***	0.67 ***	0.42 ***	1	0.79 ***	0.27 **	0.84 ***	0.81 ***	0.43 ***	0.89 ***	0.22 *	0.04	−0.18 *
Fe	0.80 ***	0.67 ***	0.46 ***	0.89 ***	1	0.24 **	0.79 ***	0.82 ***	0.48 ***	0.84 ***	0.19 *	0.08	−0.13
Li	0.41 ***	0.33 ***	0.43 ***	0.30 **	0.30 **	1	0.38 ***	0.26 **	0.29 **	0.17	0.09	−0.13	−0.14
Mg	0.87 ***	0.66 ***	0.47 ***	0.90 ***	0.89 ***	0.38 ***	1	0.88 ***	0.50 ***	0.83 ***	0.20 *	−0.01	−0.19 *
Mn	0.77 ***	0.64 ***	0.46 ***	0.87 ***	0.87 ***	0.26 **	0.91 ***	1	0.50 ***	0.83 ***	0.19 *	−0.08	−0.25 **
Ni	0.32 ***	0.27 **	0.37 ***	0.42 ***	0.38 ***	0.30 ***	0.46 ***	0.34 ***	1	0.38 ***	0.21 *	−0.02	−0.28 **
Zn	0.75 ***	0.65 ***	0.32 ***	0.85 ***	0.86 ***	0.14	0.87 ***	0.85 ***	0.31 ***	1	0.23 *	0.07	−0.19*
GPC	0.31 ***	0.22 *	0.12	0.43 ***	0.48 ***	−0.08	0.40 ***	0.47 ***	0.19 *	0.51 ***	1	−0.37 ***	−0.36 ***
GY	0.03	0.04	0.02	−0.01	−0.07	0.13	0.05	0.02	−0.03	−0.07	−0.36 ***	1	NA
GY_pTKW_	−0.11	−0.10	0.07	−0.19 *	0.22 *	0.06	−0.14	−0.15	−0.13	−0.24 **	−0.44 ***	NA	1

*, **, and *** = significant at the 0.05, 0.01, and 0.001 probability level, respectively. NA: Not Applicable.

**Table 3:** Potential candidate genes containing/flanking marker–trait associations for improving grain minerals in SHWs.

Gene Annotation (Gene ID)	Trait in Our Study ^a^	Chromosome	PVE (%) ^b^	Traits Influenced Based on the Annotations	References for the Association of Annotations with Traits
2-oxoglutarate (2OG) and Fe (II)-dependent oxygenase superfamily protein (TraesCS2A01G519900-TraesCS2A01G520000)	Cu (1)	2A	5.3	Fe, Mg	[33,40]
AP2-like ethylene-responsive transcription factor (TraesCS2A01G514200)	Cu (1)	2A	3.1	As	
ATP synthase gamma chain (TraesCS6B01G117700)	Ca (1)	6B	19.9	Fe, Zn	[41]
Chaperone protein dnaJ (TraesCS4B01G187600)	Zn (1)	4B	14.1	Cd	[32]
F-box family protein domain (TraesCS6D01G360300, TraesCS3B01G479800, TraesCS3B01G111900, TraesCS6B01G268400, TraesCS6D01G064500, TraesCS2D01G106500, TraesCS4D01G333100)	Co (1), Li (1), Mg (2), Mn (1), Zn (2)	2D, 3B, 4D, 6B, 6D	1.8–25.2	Fe	[34]
GDSL esterase/lipase (TraesCS5A01G096300)	Li (1)	5A	4.4	Fe, Zn, Mn	[42]
Kinase family protein (TraesCS1B01G375400)	Li (1)	1B	13.5	Cd, Zn	[35,36]
Leucine rich receptor-like protein kinase (TraesCS2D01G466400, TraesCS6B01G384300-TraesCS6B01G384400, TraesCS4A01G490700, TraesCS3B01G192500)	Ca (1), Li (1), Mg, Zn	2D, 3B, 4A, 6B	1.8–12.6	Fe	[35,36,42]
MYB transcription factor (TraesCS6B01G053100)	Ca (1)	6B	9.9	Cd, Fe, Zn	[37]
Na-translocating NADH-quinone reductase subunit A (TraesCS1A01G432900)	Fe (1)	1A	11.2	Fe	[38]
No apical meristem (NAM) protein (TraesCS7A01G068200)	Ca (1)	7A	11.8	Fe, Zn, N	[42]
Peroxidase (TraesCS6A01G081700)	Ca (1)	6A	9	Cd	[43]
Phosphate translocator (TraesCS3B01G192400)	Zn (1)	3B	1.8	P	[44]
Potassium transporter (TraesCS2D01G106600)	Mn (1)	2D	8.7	K	[45]
Protein COBRA, putative (TraesCS4B01G187300)	Mn (1)	4B	13.4	Al	[36]
Protein DETOXIFICATION (TraesCS3A01G300400)	Mg (1)	3A	14.6	Fe	[46]
Protein ROOT HAIR DEFECTIVE 3 homolog (TraesCS1A01G003300-TraesCS1A01G003400)	Zn (1)	1A	3	Cd	[32]
ROP guanine nucleotide exchange factor 10 (TraesCS4D01G333000)	Mg (1)	4D	7.9	Fe	[36]
Universal stress protein family (TraesCS3B01G418000)	Ca (1)	3B	2.9	Fe, Zn	[37]

^a^ The count of marker–trait associations (in the parenthesis) for either single or multiple traits located within genes that have the same gene annotation; ^b^ PVE, phenotypic variance explained by the MTA.

## Supplementary Materials

Supplementary materials can be found at http://www.mdpi.com/1422-0067/19/10/3237/s1.

## Abbreviations

BLUP	Best Linear Unbiased Predictor
Ca	Calcium
Cd	Cadmium
Co	Cobalt
Cu	Copper
Fe	Iron
GBS	Genotyping-By-Sequencing
GPC	Grain Protein Concentration
GWAS	Genome-Wide Association Study
GY	Grain Yield
Li	Lithium
MAF	Minor Allele Frequency
Mg	Magnesium
Mn	Manganese
MTA	Marker-Trait Association
Ni	Nickel
PVE	Phenotypic Variance Explained
QTL	Quantitative Trait Loci
SHW	Synthetic Hexaploid Wheat
SNP	Single Nucleotide Polymorphism
TKW	Thousand Kernel Weight
Zn	Zinc
