# Supplementary material for: Genome-Wide Association Study Reveals Novel Genomic Regions Associated with 10 Grain Minerals in Synthetic Hexaploid Wheat
Source: Int J Mol Sci. 2018 Oct 19;19(10):3237. doi: 10.3390/ijms19103237 (PMC6214031; doi:10.3390/ijms19103237)
Supplement: Supplementary file 1 [file ijms-19-03237-s001.zip › ijms-368117-sup/Supplementaryfiles/Figure S1.docx]

**A genome-wide association study reveals novel genomic regions associated with 10 grain minerals in synthetic hexaploid wheat**

**Bhatta et al. 2018. * Correspondence:** Corresponding Author: [a.morgounov@cgiar.org](mailto:a.morgounov@cgiar.org)

**Figure S1**. Manhattan and quantile-quantile plots obtained from the genome-wide association study for 10-grain minerals in 123 synthetic hexaploid wheat germplasm.


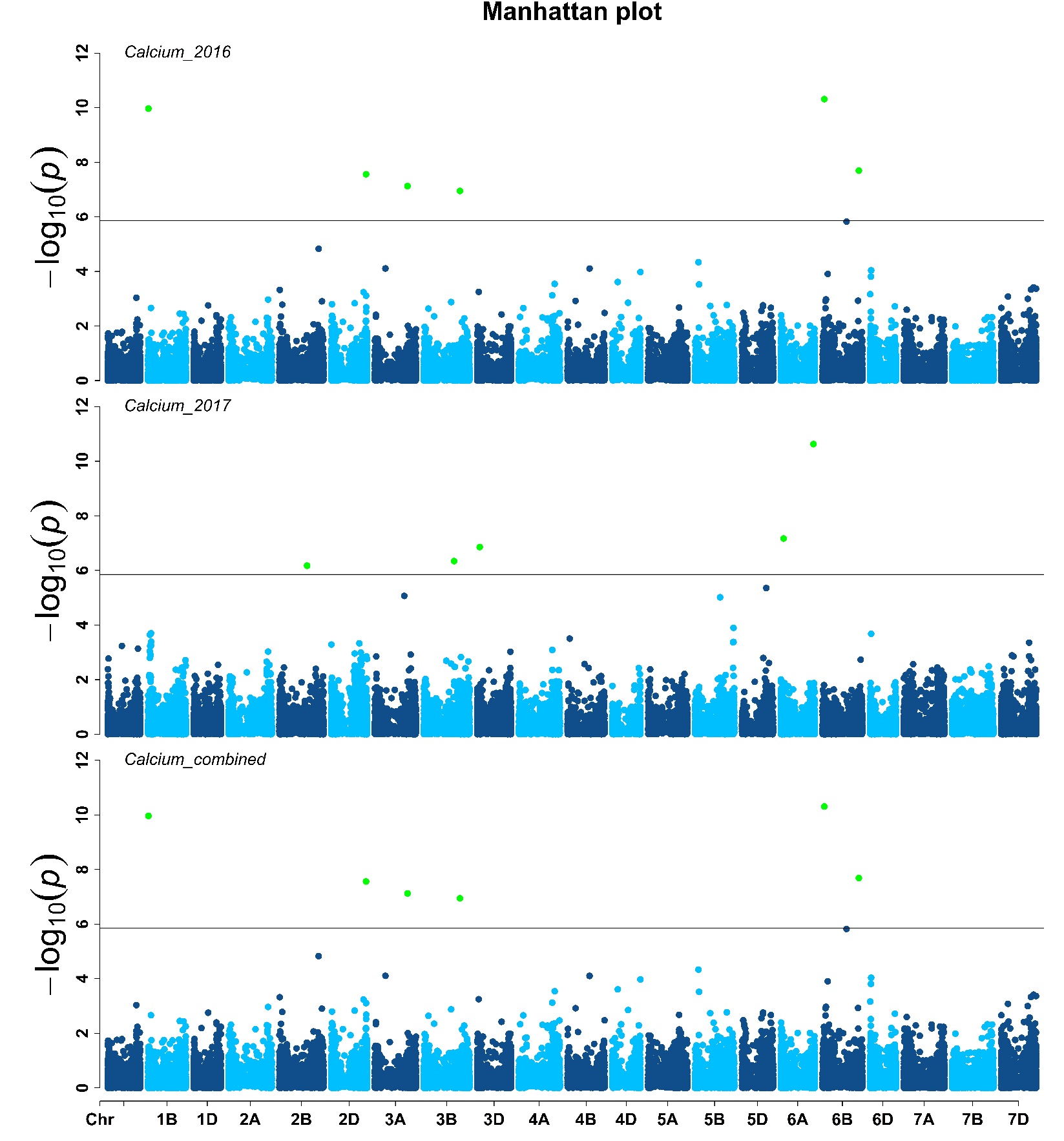


**Calcium**. Manhattan plot for Ca in 123 synthetic hexaploid wheats based on best linear unbiased prediction values. The solid black line showed the expected value at Bonferroni correction at 5% level of significance [-log10(P)=5.85].


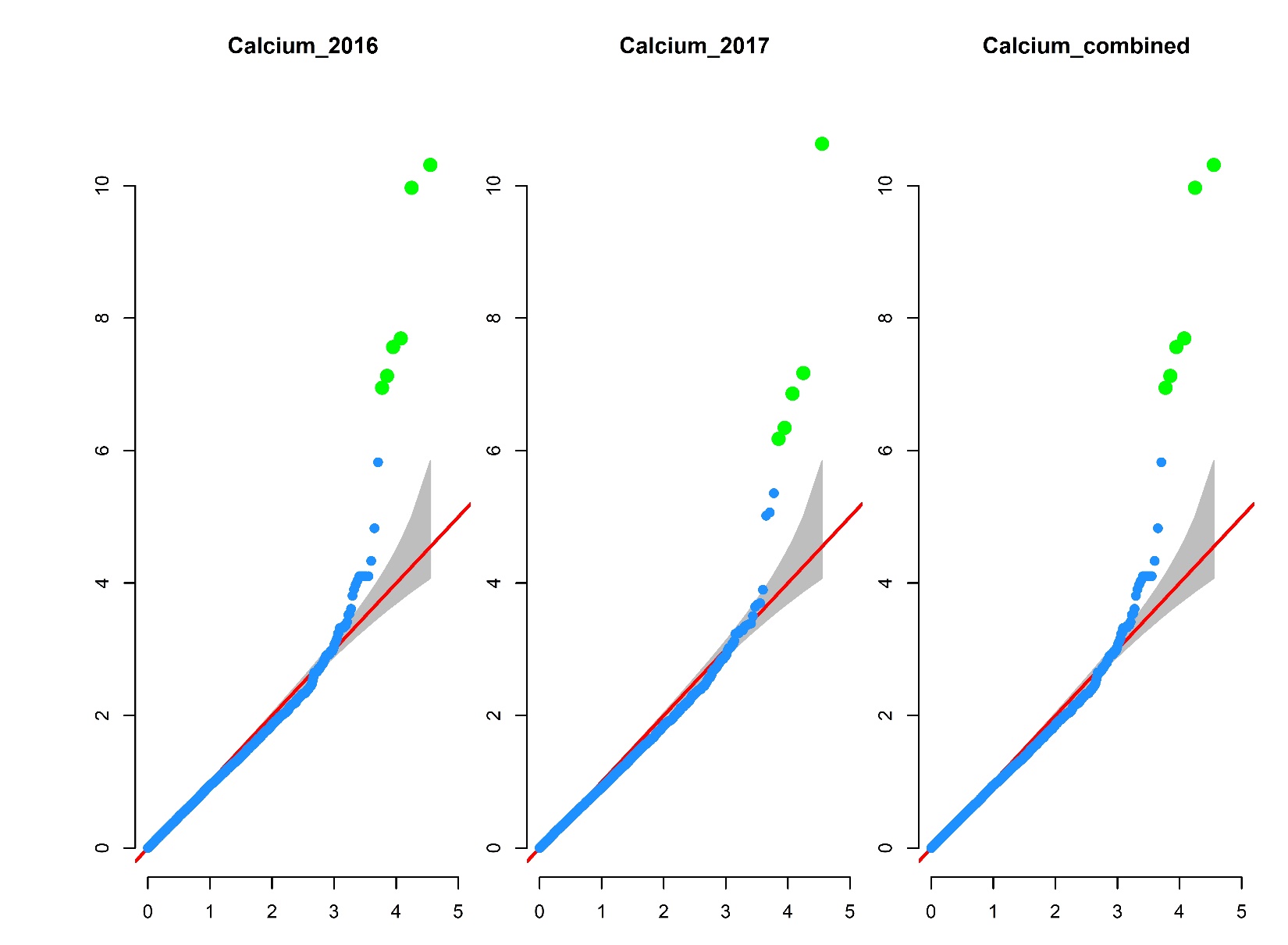
**Calcium.** Quantile-quantile plot for Ca in 123 synthetic hexaploid wheats based on best linear unbiased prediction values. The green dot showed the expected value at Bonferroni correction at 5% level of significance [-log10(P)=5.85].


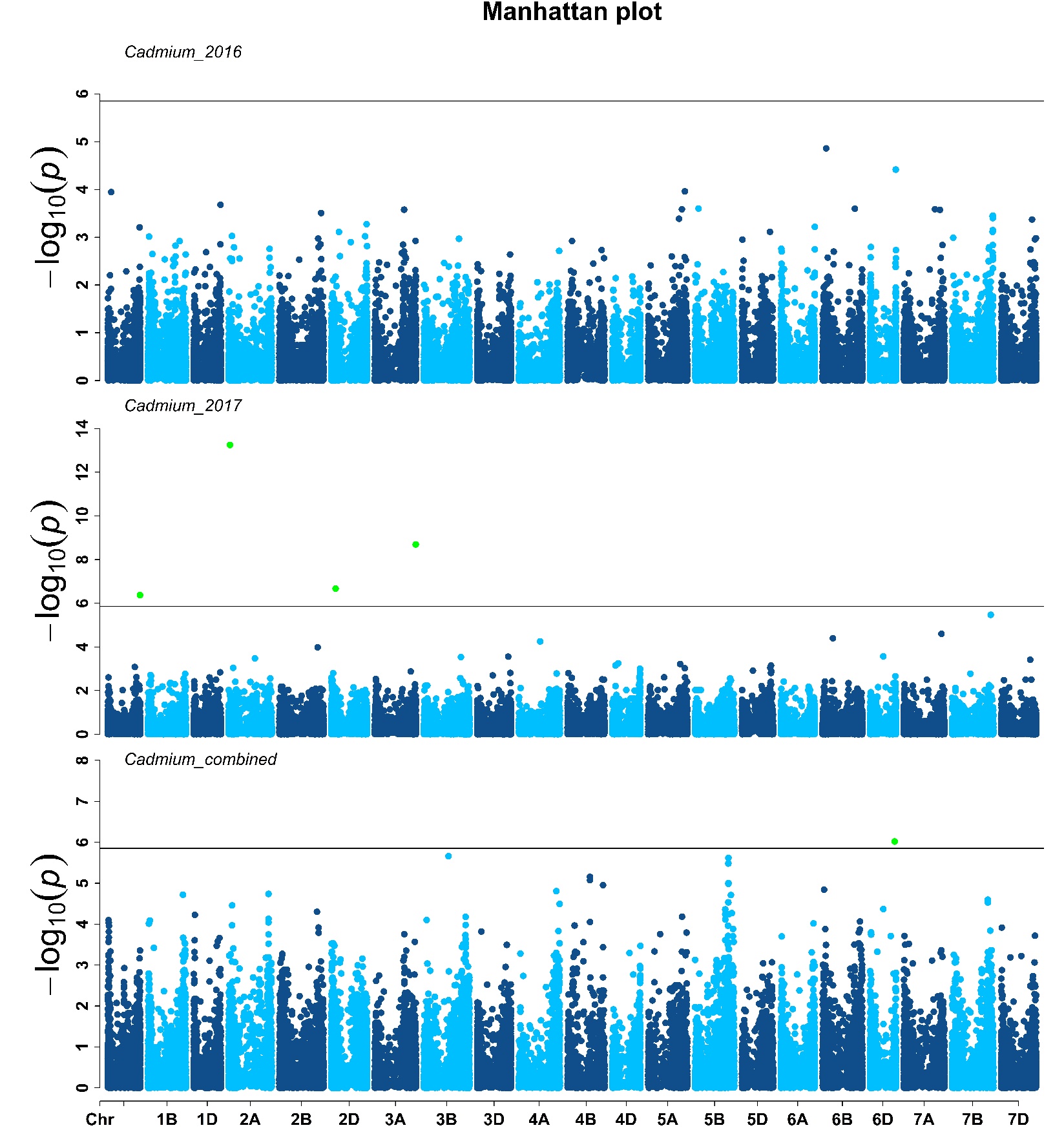


**Cadmium.** Manhattan plot for Cd in 123 synthetic hexaploid wheats based on best linear unbiased prediction values. The green dot showed the expected value at Bonferroni correction at 5% level of significance [-log10(P)=5.85].


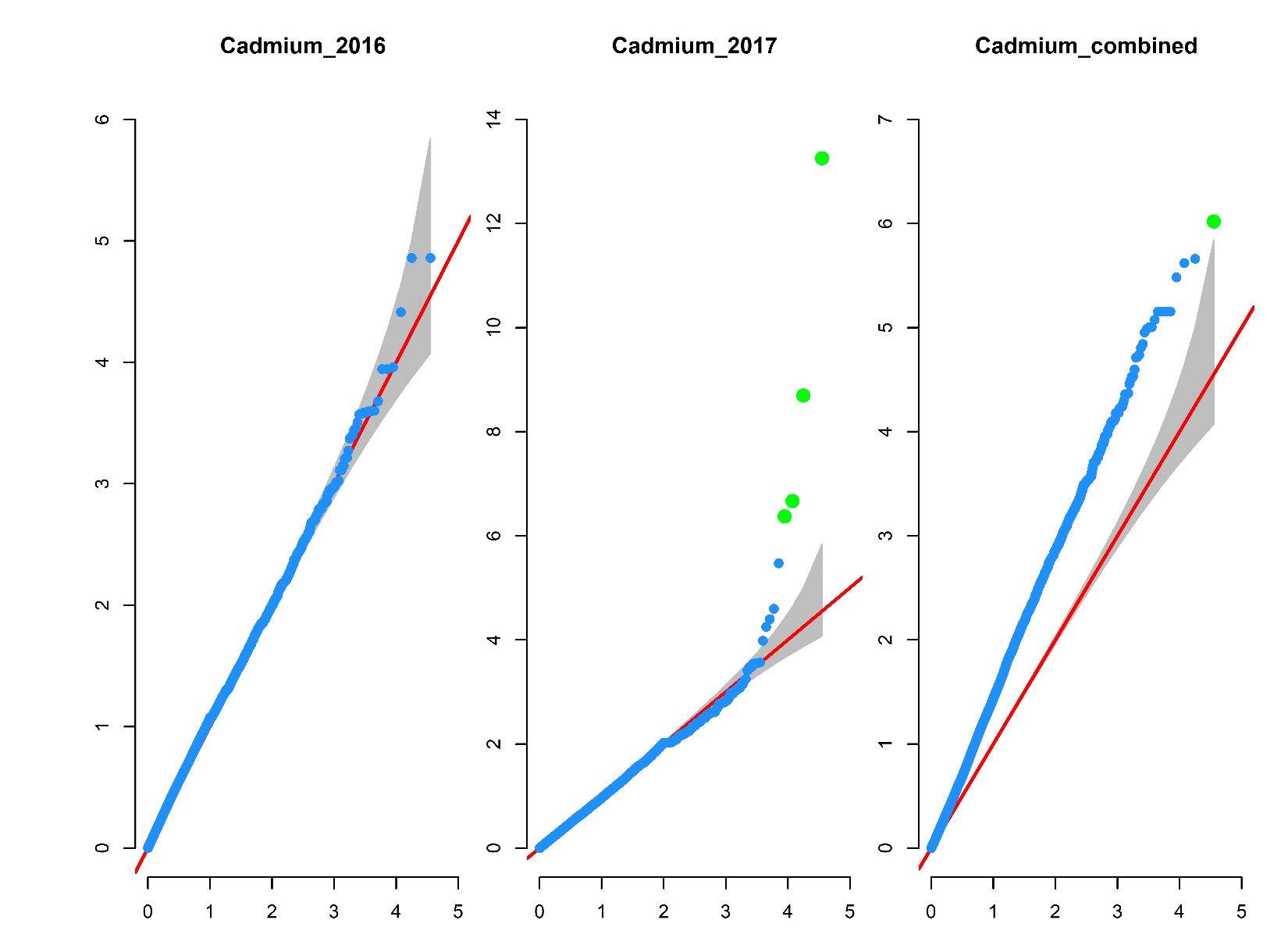


**Cadmium.** Quantile-quantile plot for Cd in 123 synthetic hexaploid wheats based on best linear unbiased prediction values. The green dot showed the expected value at Bonferroni correction at 5% level of significance [-log10(P)=5.85]. Note: Only Cadmium_2017 result was taken into consideration based on fit of the Q-Q plot.


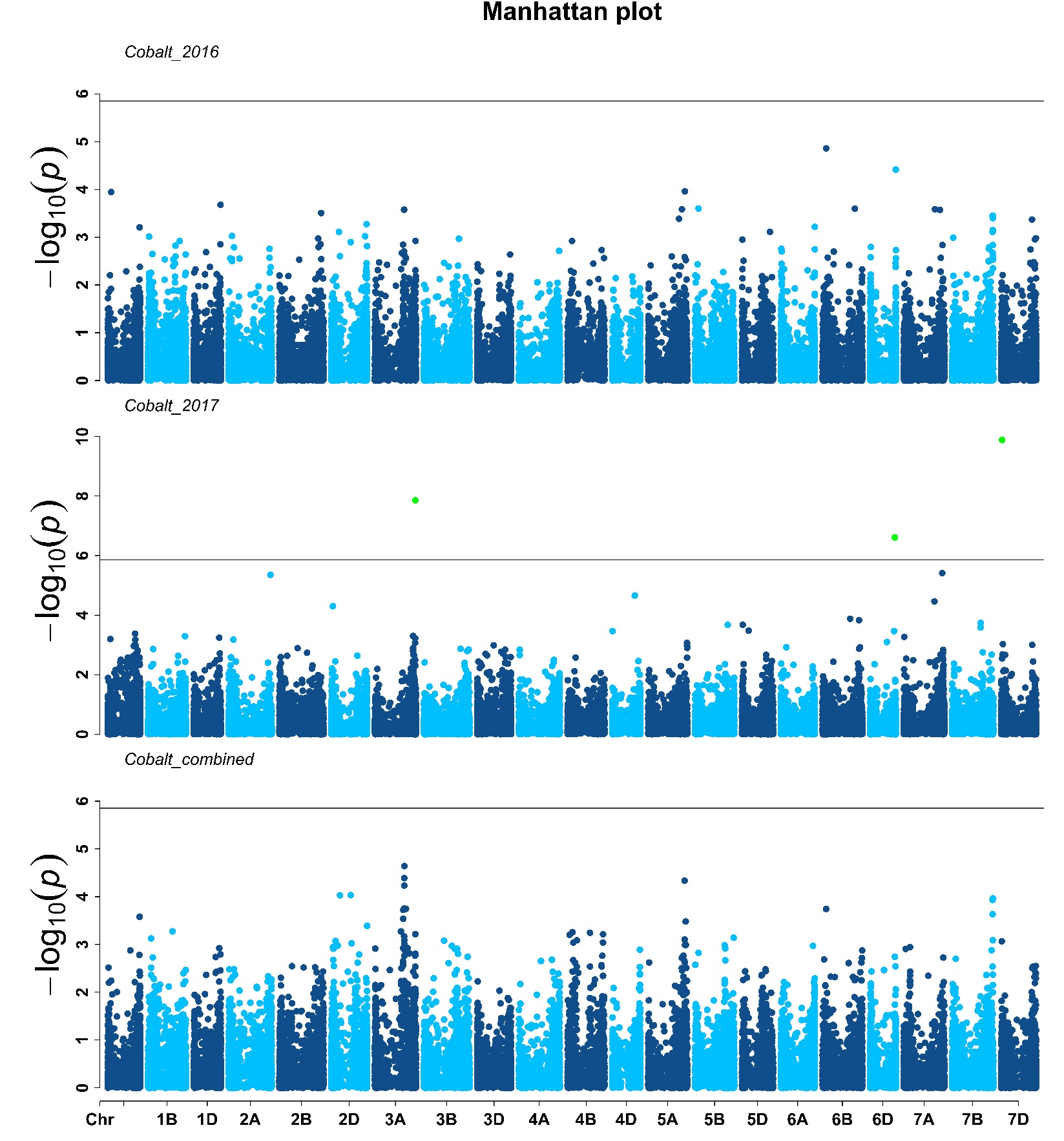
**Cobalt.** Manhattan plot for Co in 123 synthetic hexaploid wheats based on best linear unbiased prediction values. The solid black line showed the expected value at Bonferroni correction at 5% level of significance [-log10(P)=5.85].


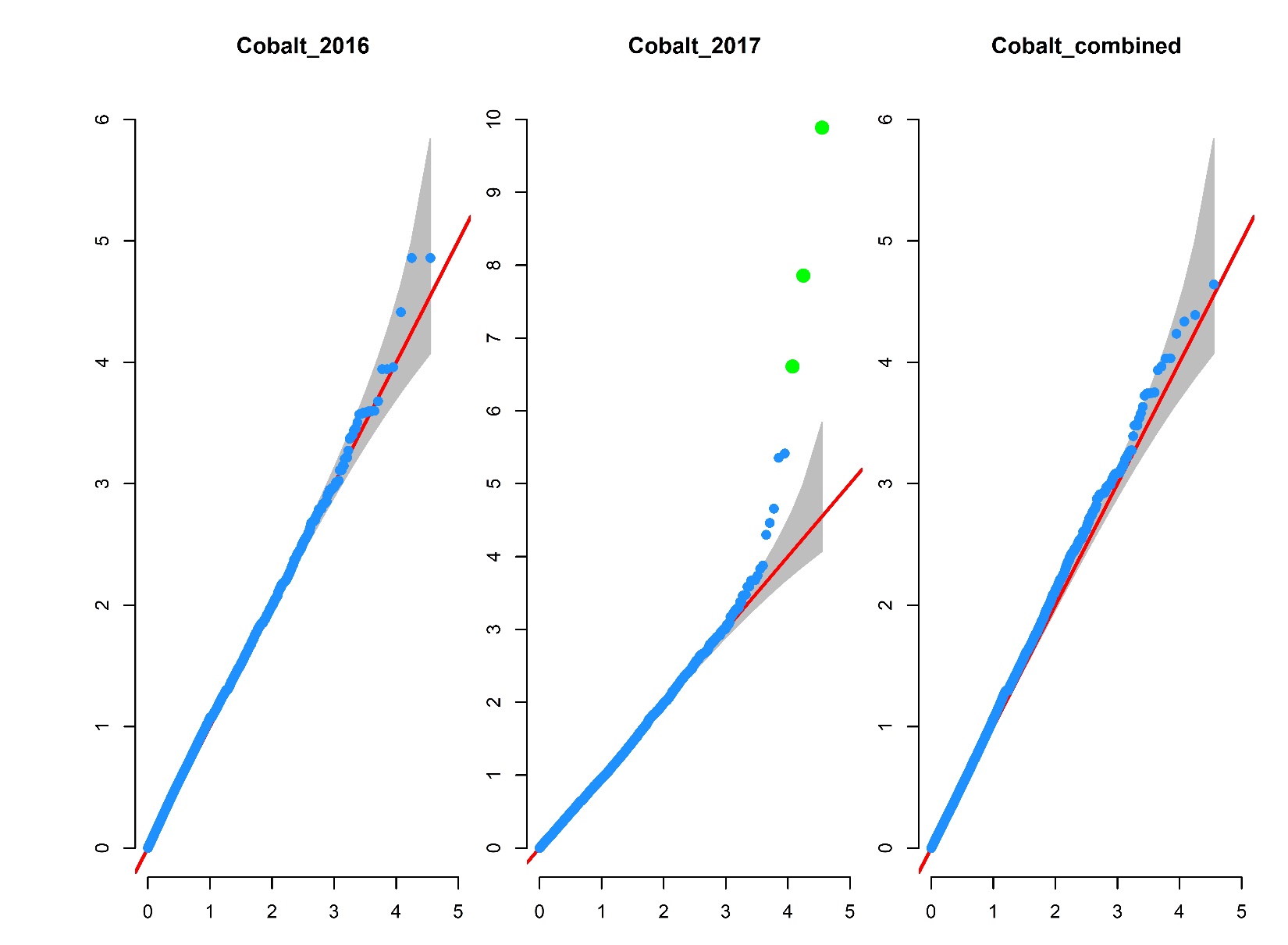
**Cobalt.** Quantile-quantile plot for Co in 123 synthetic hexaploid wheats based on best linear unbiased prediction values. The green dot showed the expected value at Bonferroni correction at 5% level of significance [-log10(P)=5.85]. Note: Only Cobalt_2017 result was taken into consideration based on fit of the Q-Q plot.


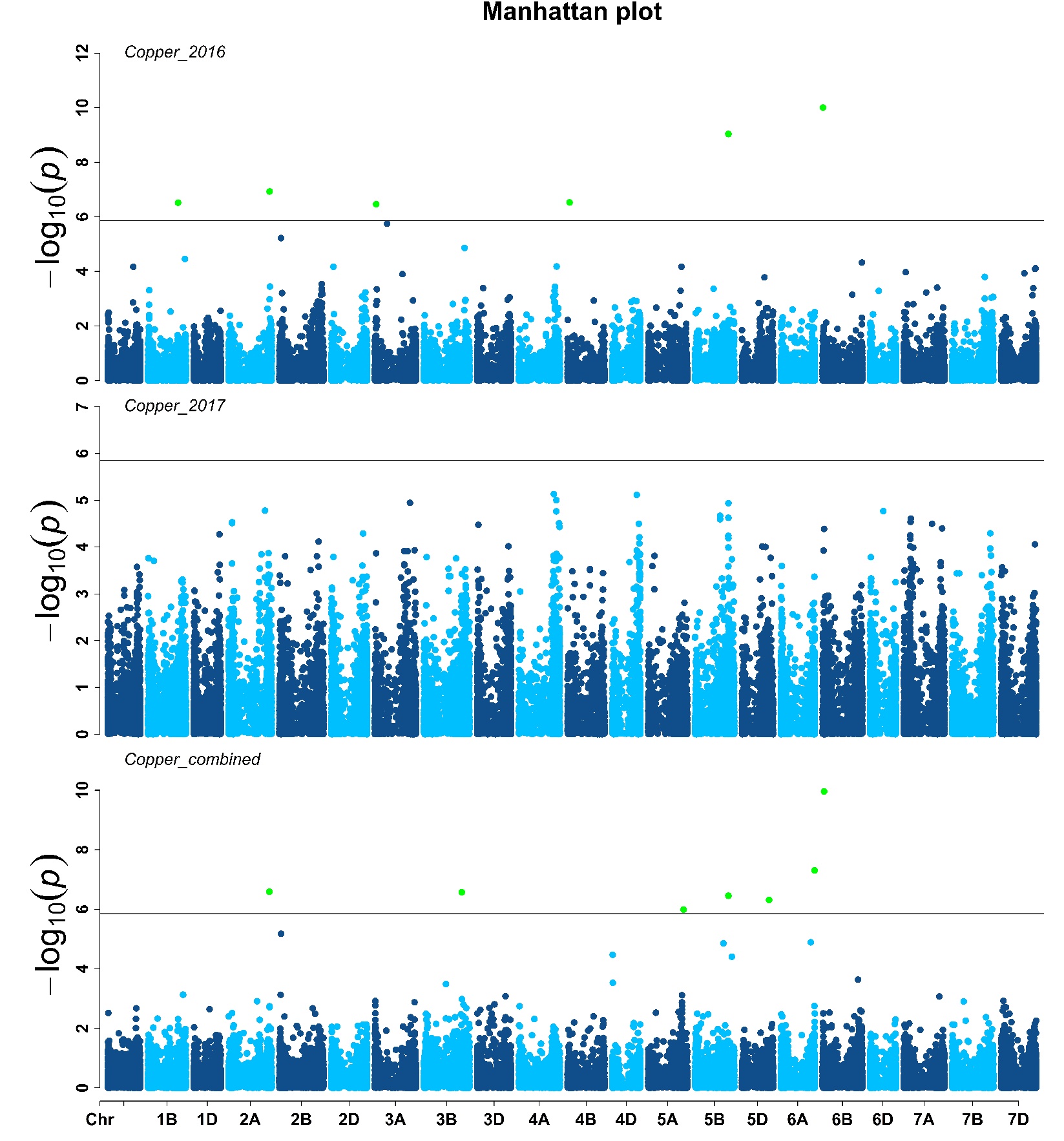
**Copper.** Manhattan plot for Cu in 123 synthetic hexaploid wheats based on best linear unbiased prediction values. The solid black line showed the expected value at Bonferroni correction at 5% level of significance [-log10(P)=5.85].


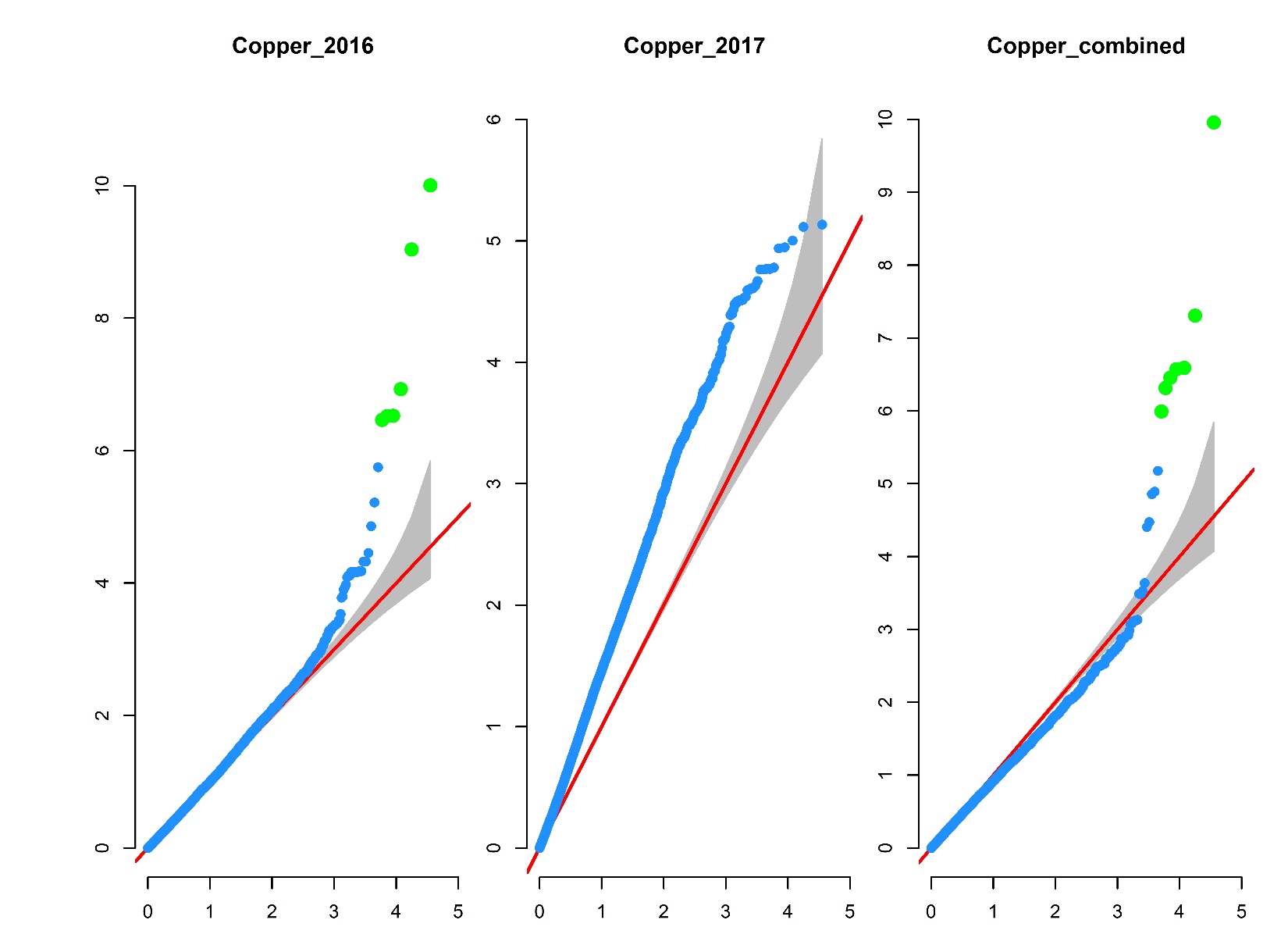
**Copper.** Quantile-quantile plot for Cu in 123 synthetic hexaploid wheats based on best linear unbiased prediction values. The green dot showed the expected value at Bonferroni correction at 5% level of significance [-log10(P)=5.85]. Note: Only Copper_2016 and Copper_combined results were taken into consideration based on fit of the Q-Q plot.


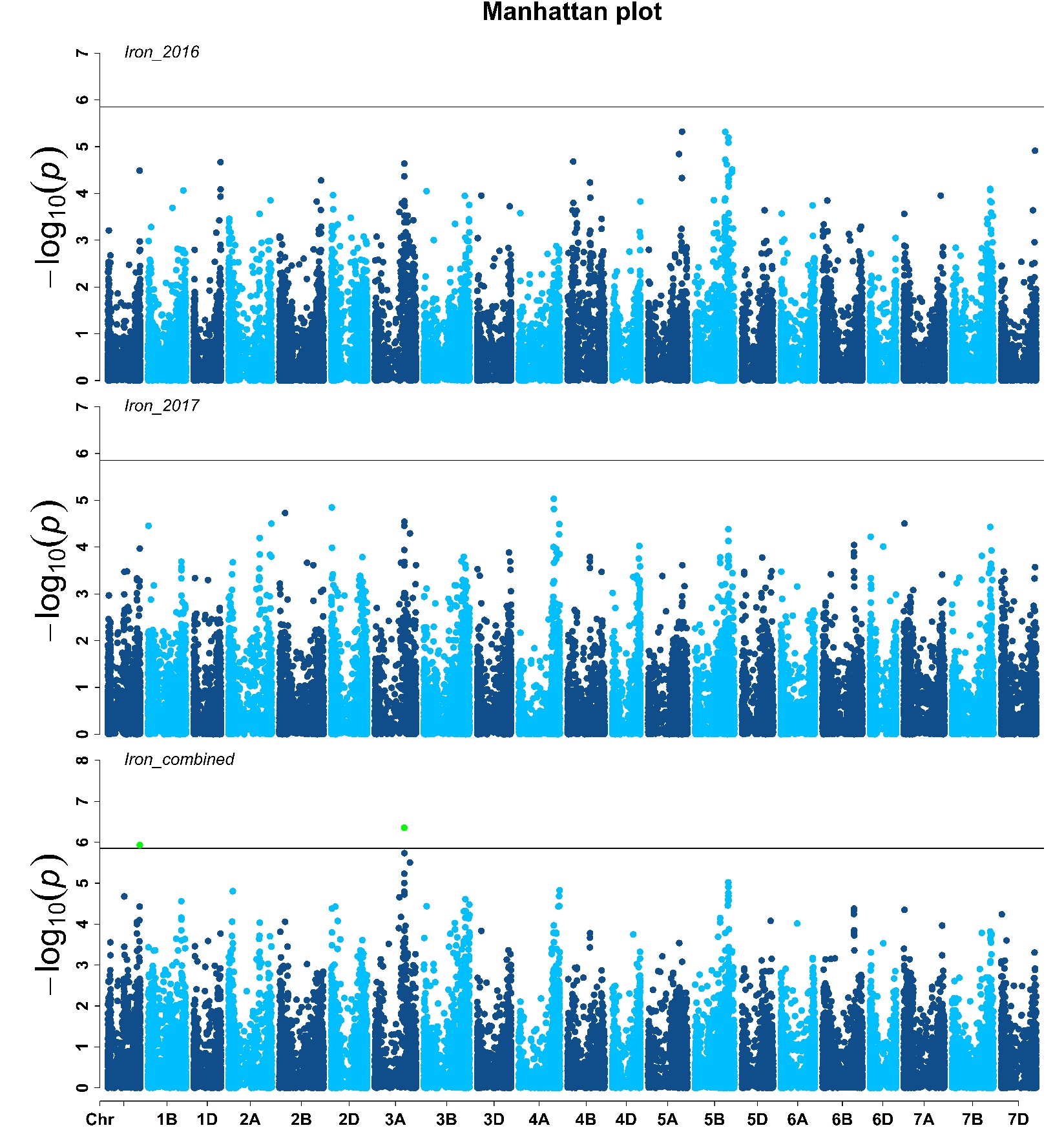
**Iron.** Manhattan plot for Fe in 123 synthetic hexaploid wheats based on best linear unbiased prediction values. The solid black line showed the expected value at Bonferroni correction at 5% level of significance [-log10(P)=5.85].

**
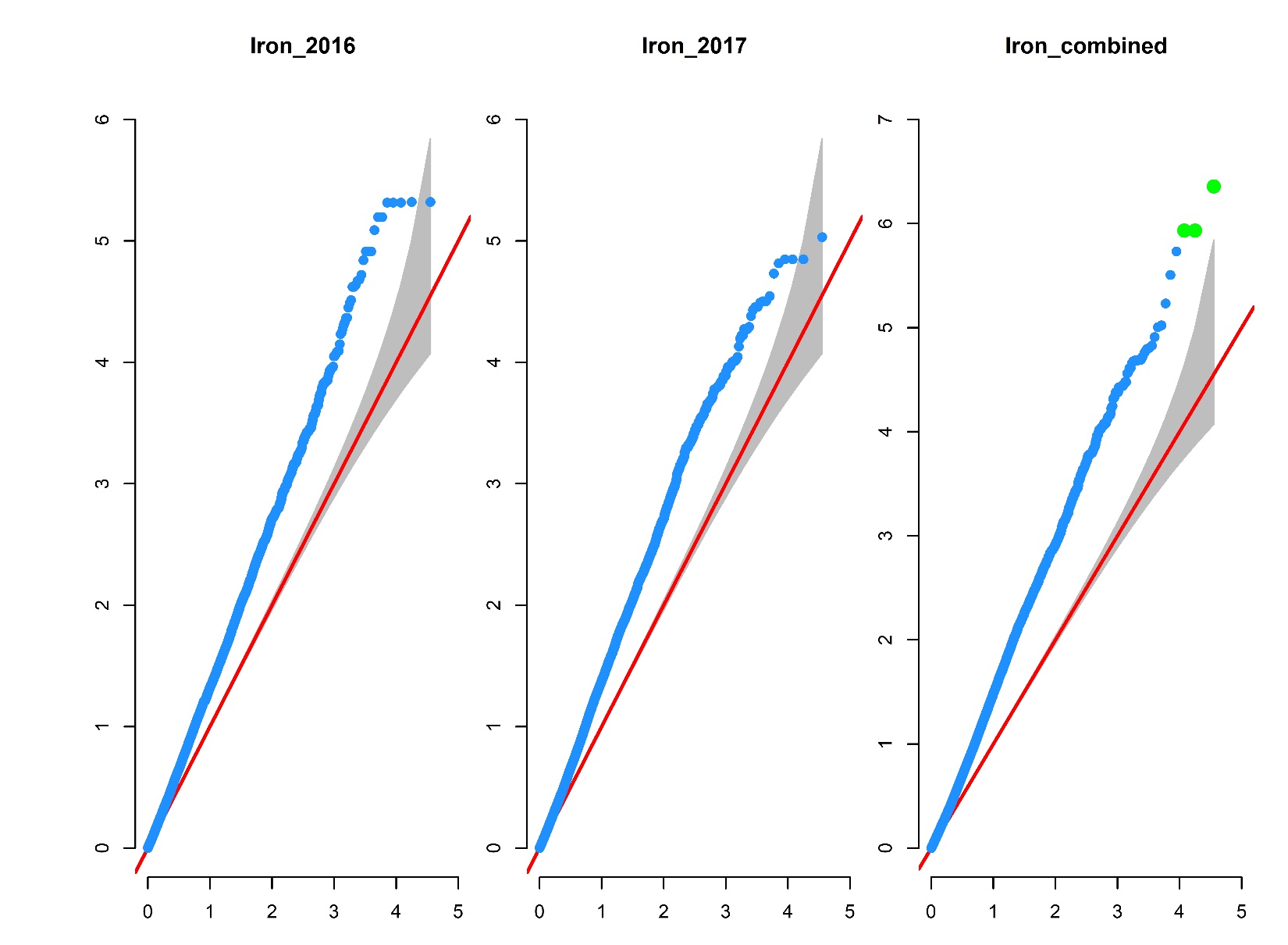
Iron.** Quantile-quantile plot for Fe in 123 synthetic hexaploid wheats based on best linear unbiased prediction values. The green dot showed the expected value at Bonferroni correction at 5% level of significance [-log10(P)=5.85].


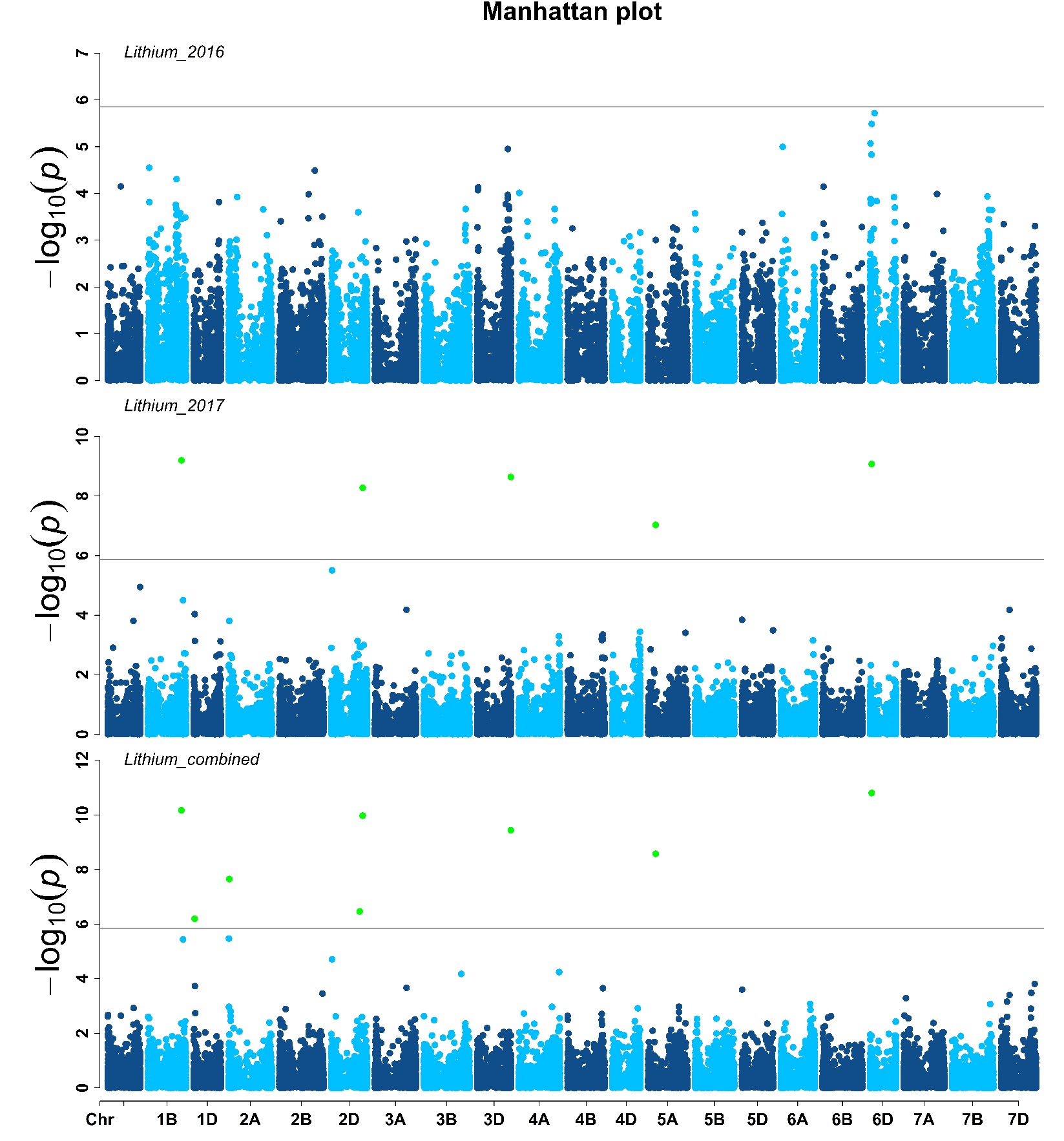


**Lithium.** Manhattan plot for Li in 123 synthetic hexaploid wheats based on best linear unbiased prediction values. The solid black line showed the expected value at Bonferroni correction at 5% level of significance [-log10(P)=5.85].


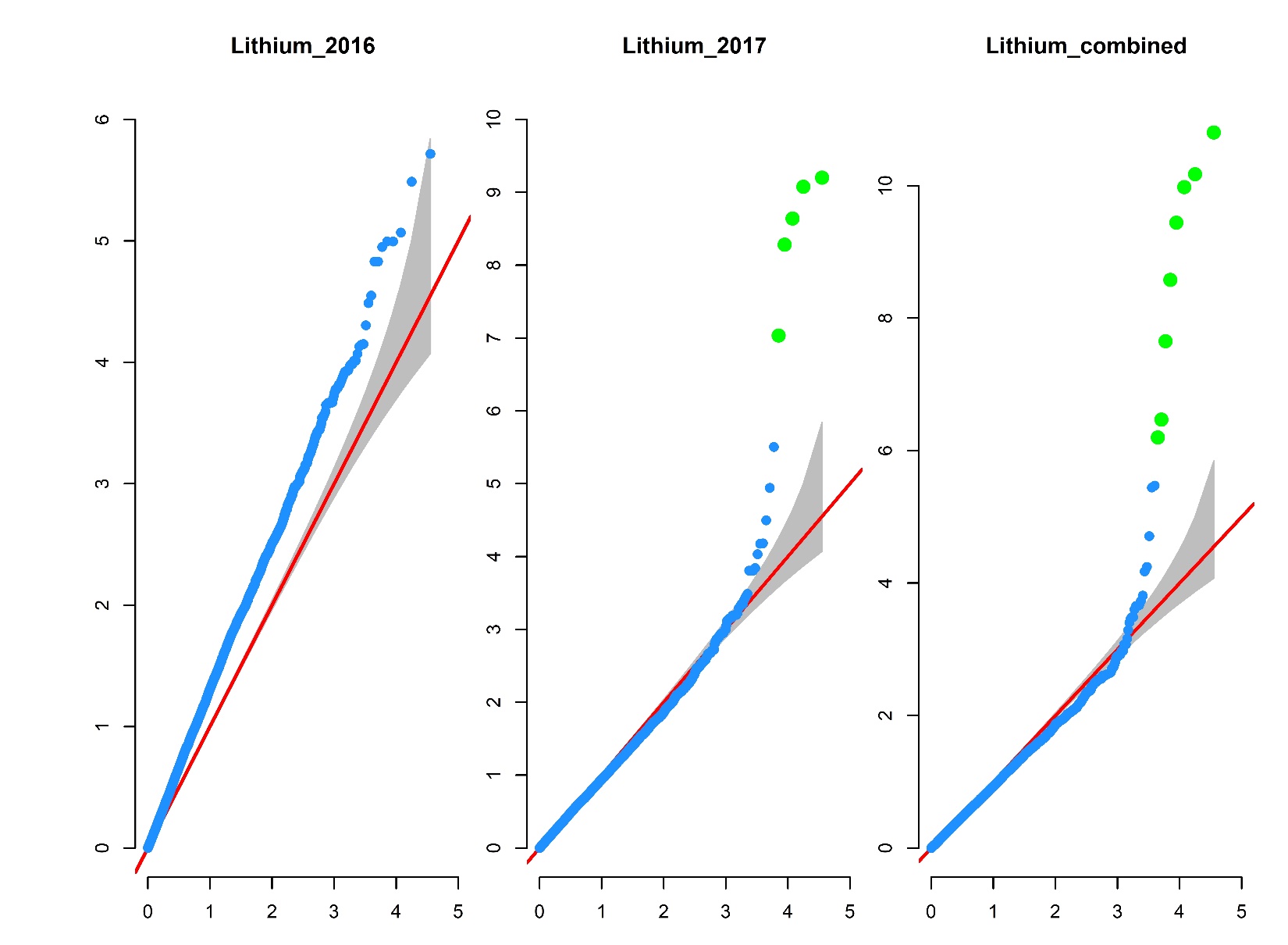
**Lithium.** Quantile-quantile plot for Li in 123 synthetic hexaploid wheats based on best linear unbiased prediction values. The green dot showed the expected value at Bonferroni correction at 5% level of significance [-log10(P)=5.85]. Note: Only Lithium_2017 and Lithium_combined results were taken into consideration based on fit of the Q-Q plot.


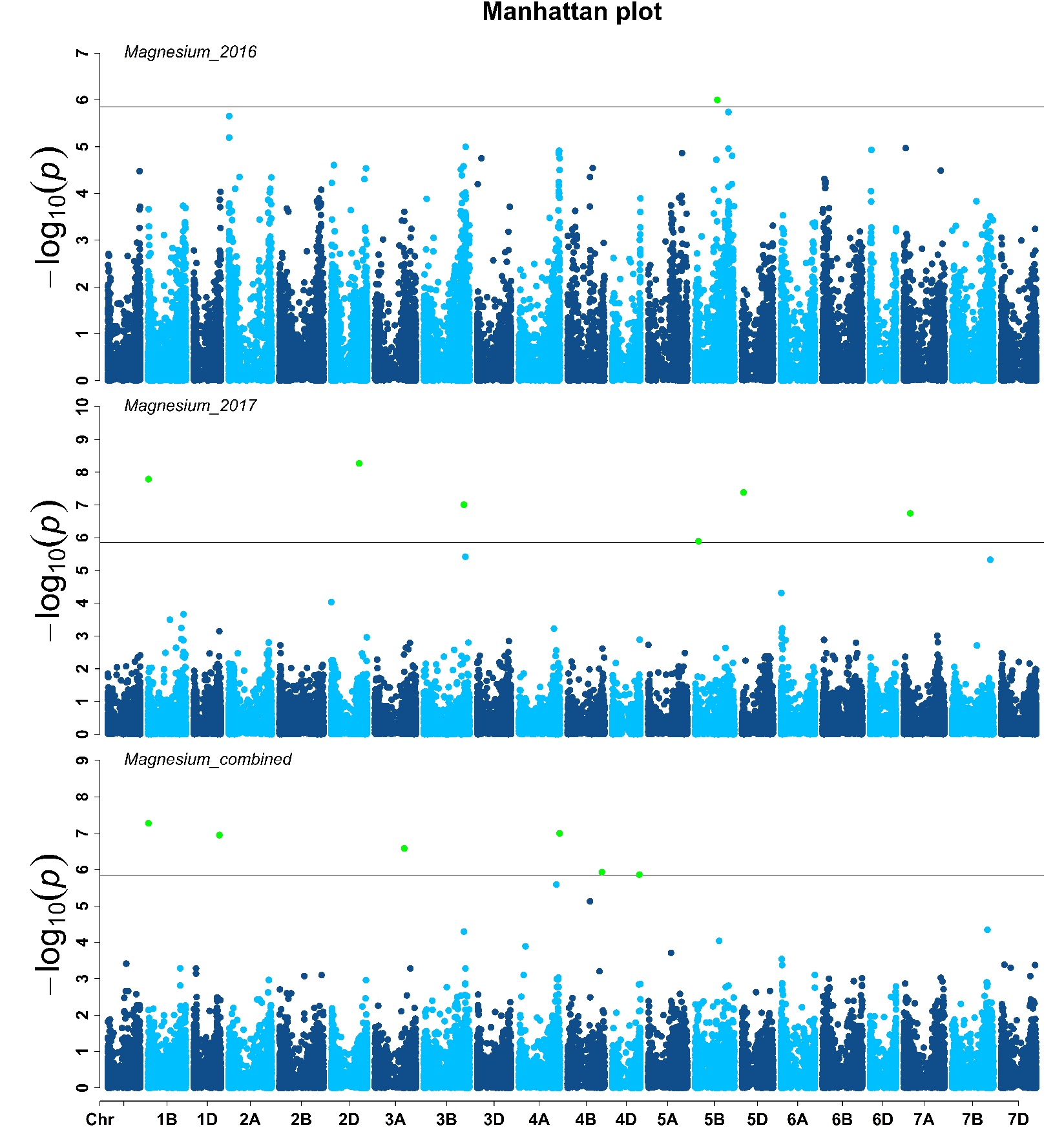
**Magnesium.** Manhattan plot for Mg in 123 synthetic hexaploid wheats based on best linear unbiased prediction values. The solid black line showed the expected value at Bonferroni correction at 5% level of significance [-log10(P)=5.85].


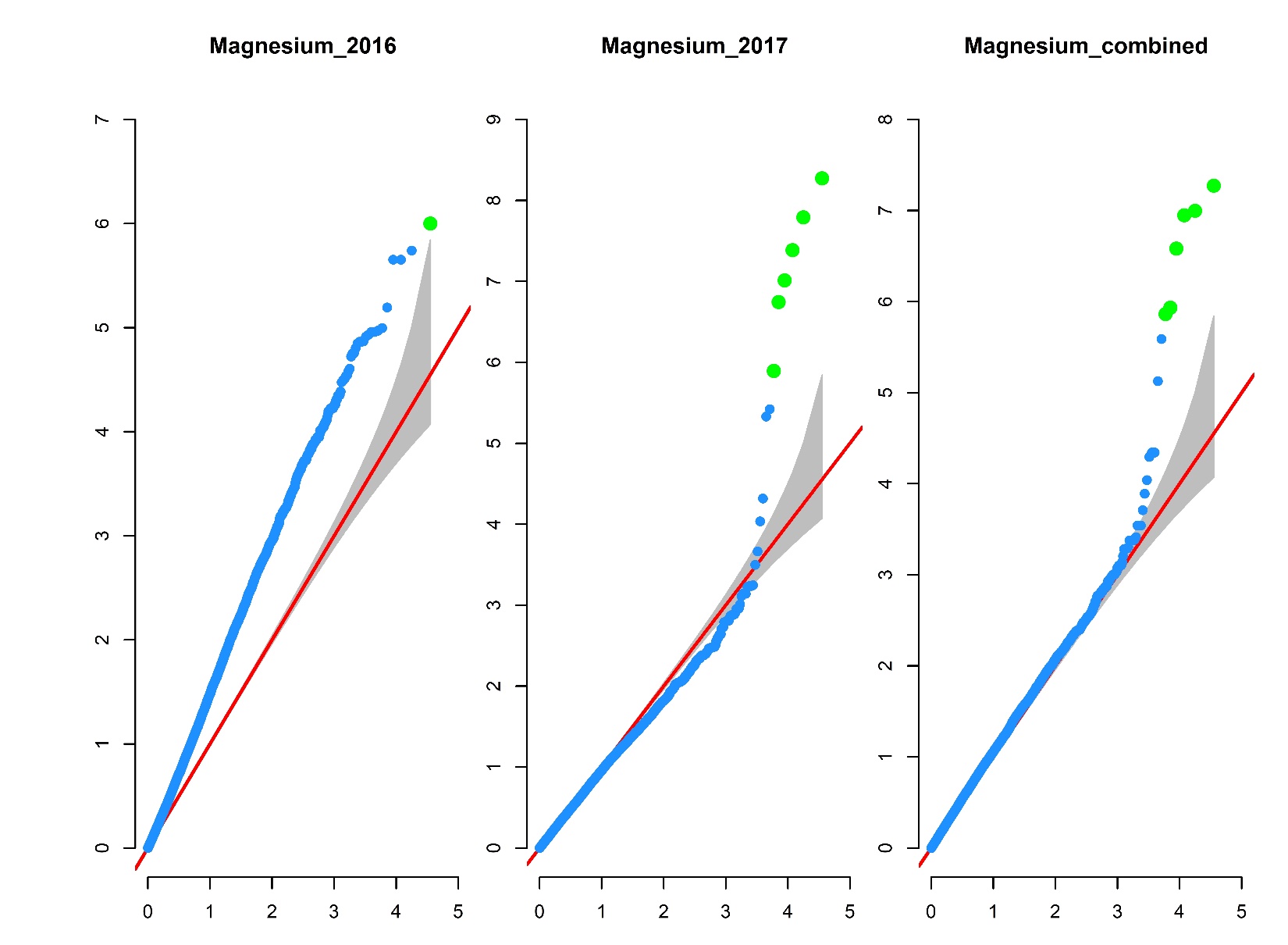
**Magnesium.** Quantile-quantile plot for Mg in 123 synthetic hexaploid wheats based on best linear unbiased prediction values. The green dot showed the expected value at Bonferroni correction at 5% level of significance [-log10(P)=5.85]. Note: Only Magnesium_2017 and Magnesium_combined results were taken into consideration based on fit of the Q-Q plot.


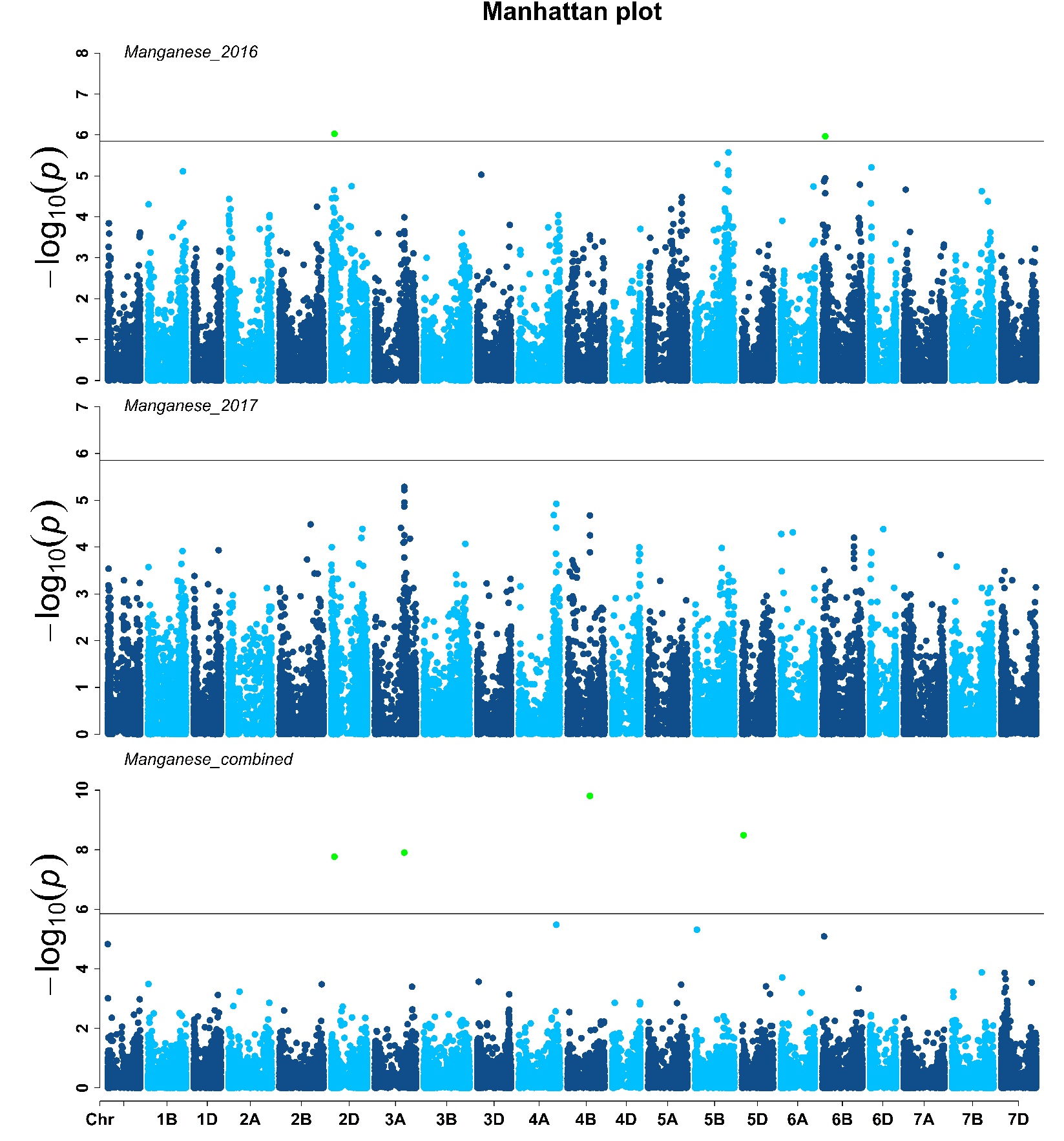
**Manganese.** Manhattan plot for Mn in 123 synthetic hexaploid wheats based on best linear unbiased prediction values. The solid black line showed the expected value at Bonferroni correction at 5% level of significance [-log10(P)=5.85].


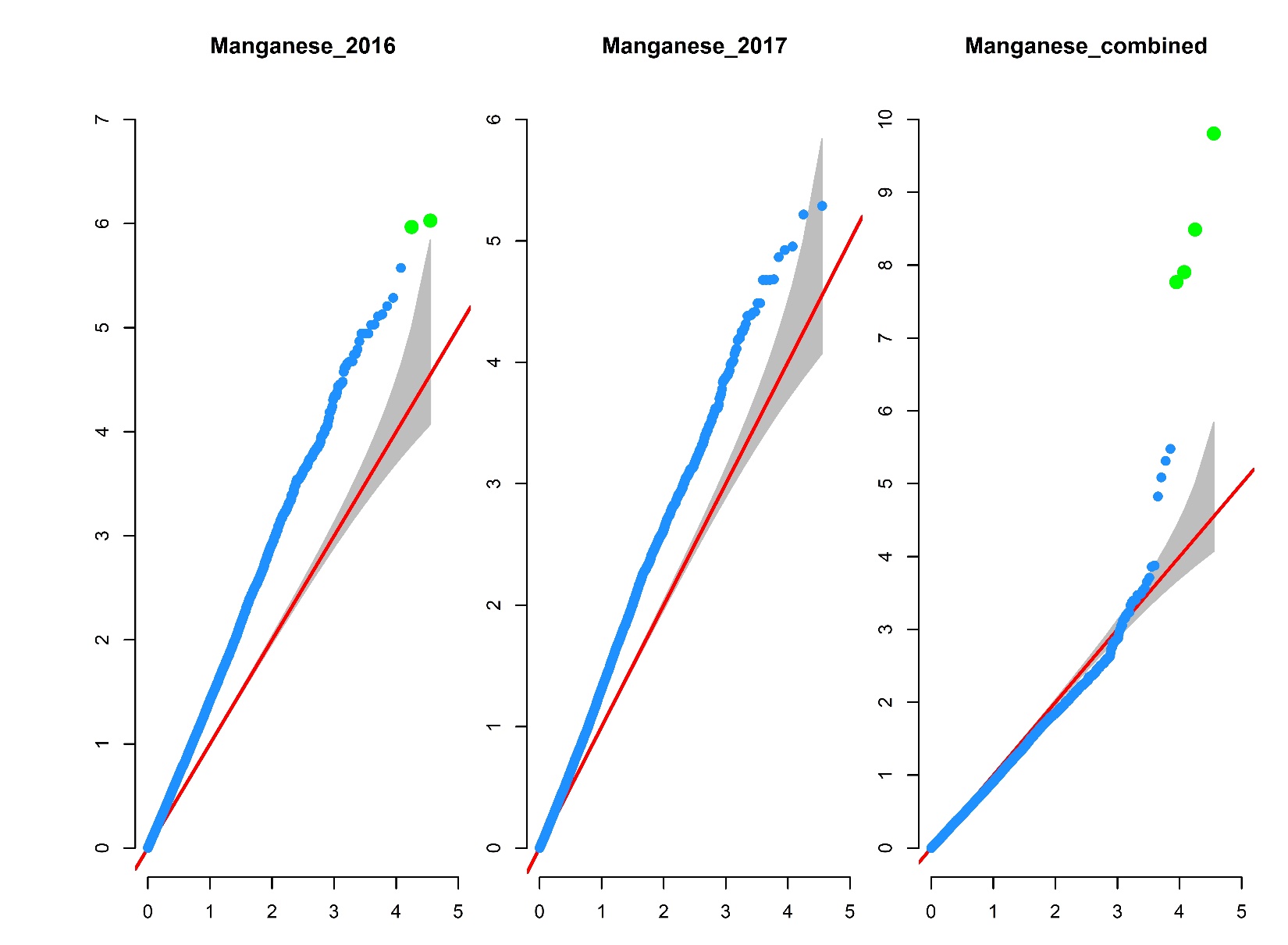
**Manganese.** Quantile-quantile plot for Mn in 123 synthetic hexaploid wheats based on best linear unbiased prediction values. The green dot showed the expected value at Bonferroni correction at 5% level of significance [-log10(P)=5.85]. Note: Only Manganese_combined result was taken into consideration based on fit of the Q-Q plot.

**
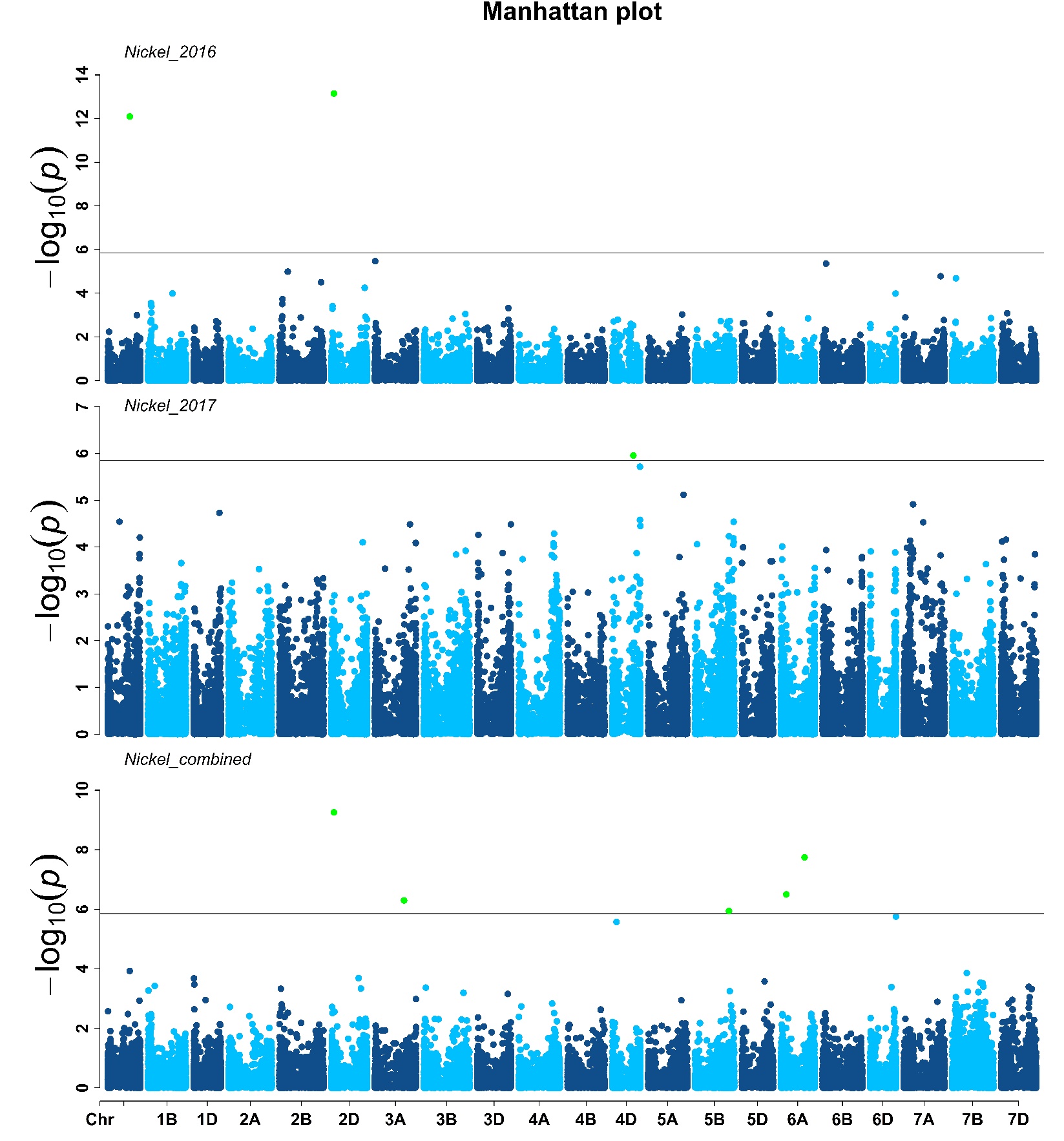
Nickel.** Manhattan plot for Ni in 123 synthetic hexaploid wheats based on best linear unbiased prediction values. The solid black line showed the expected value at Bonferroni correction at 5% level of significance [-log10(P)=5.85].


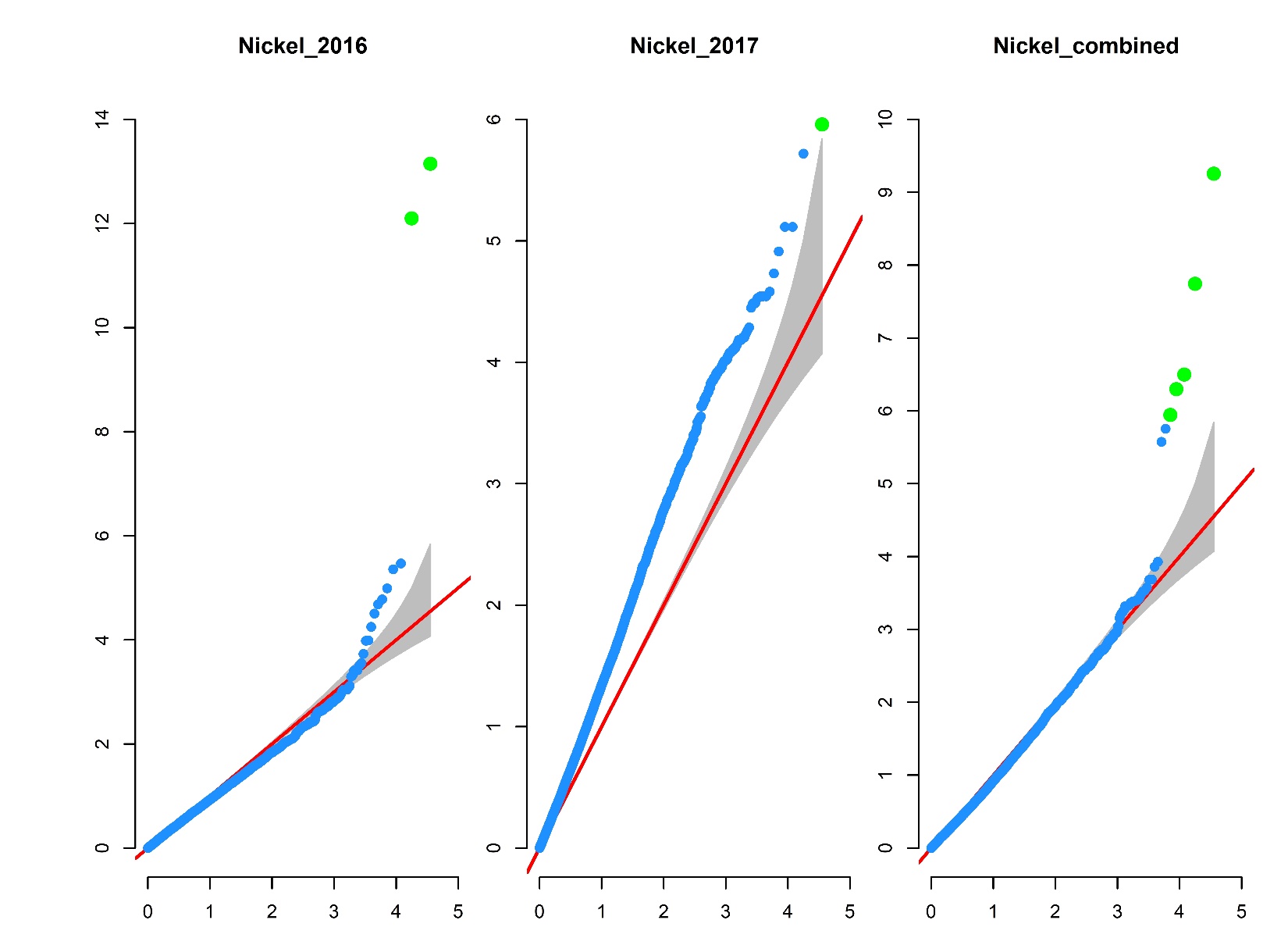
**Nickel.** Quantile-quantile plot for Ni in 123 synthetic hexaploid wheats based on best linear unbiased prediction values. The solid black line showed the expected value at Bonferroni correction at 5% level of significance [-log10(P)=5.85]. Note: Only Nickel_2016 and Nickel_combined results were taken into consideration based on fit of the Q-Q plot.


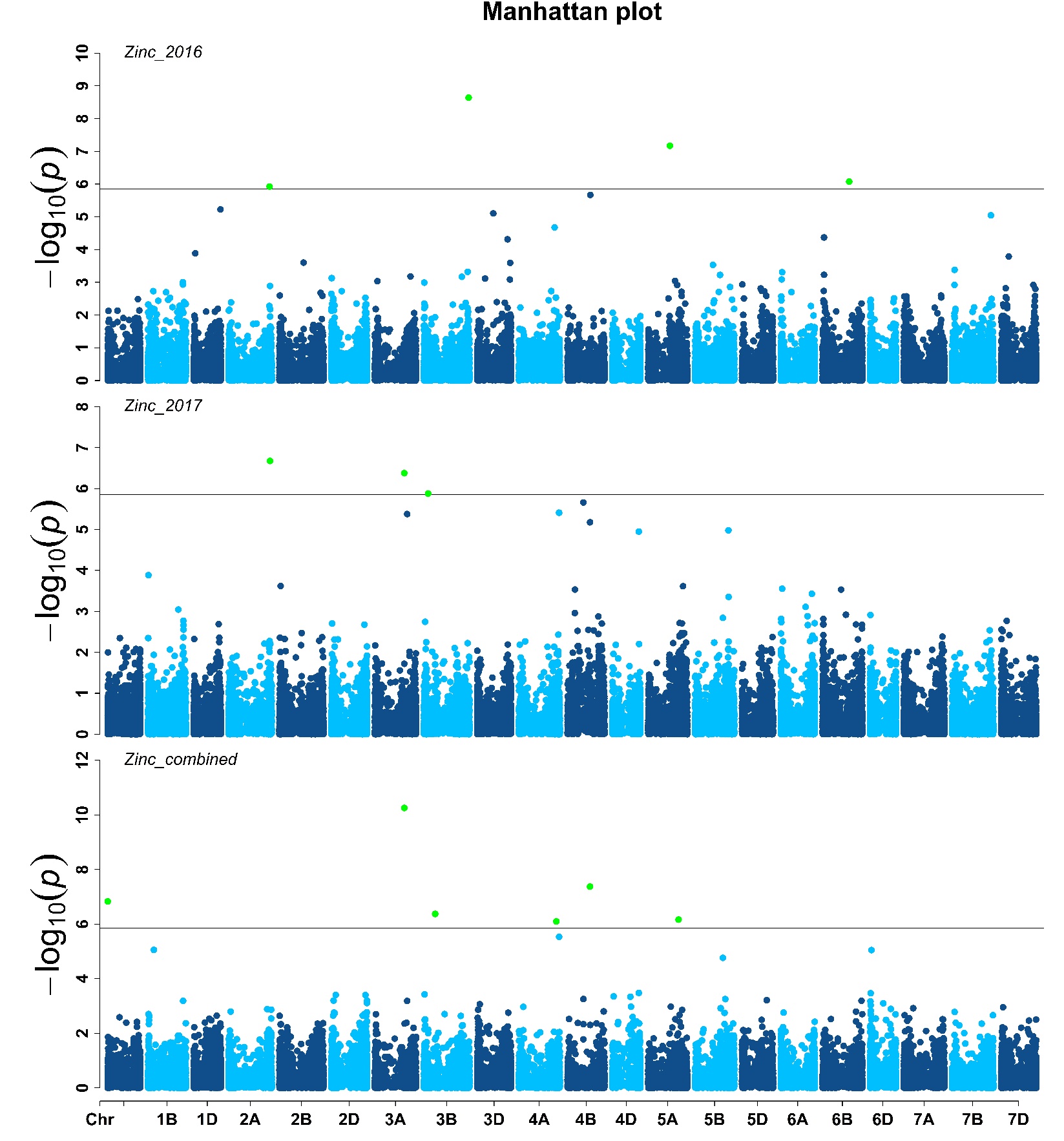
**Zinc.** Manhattan plot for Zn in 123 synthetic hexaploid wheats based on best linear unbiased prediction values. The solid black line showed the expected value at Bonferroni correction at 5% level of significance [-log10(P)=5.85].


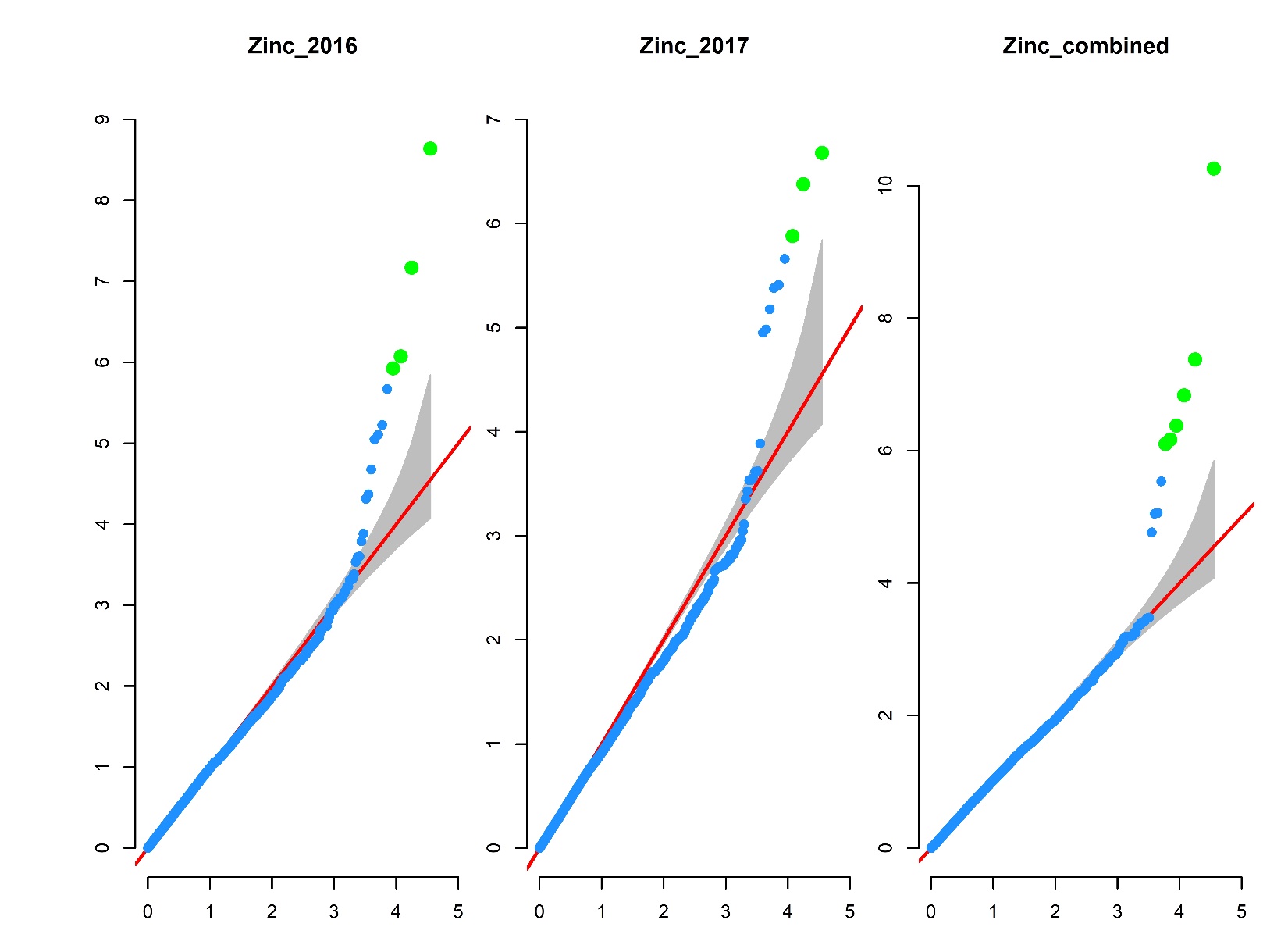
**Zinc.** Quantile-quantile plot for Zn in 123 synthetic hexaploid wheats based on best linear unbiased prediction values. The solid black line showed the expected value at Bonferroni correction at 5% level of significance [-log10(P)=5.85].
