# Supplementary material for: Genome-Wide Association Study Reveals Novel Genomic Regions Associated with 10 Grain Minerals in Synthetic Hexaploid Wheat
Source: Int J Mol Sci. 2018 Oct 19;19(10):3237. doi: 10.3390/ijms19103237 (PMC6214031; doi:10.3390/ijms19103237)
Supplement: Supplementary file 1 [file ijms-19-03237-s001.zip › ijms-368117-sup/Supplementaryfiles/Table S5.docx]

**Table S5**. Soil sample analysis in 2016 and 2017 growing season in Konya, Turkey^a^

| Year | Sample | pH | Electrical conductivity | P_2_0_5_ | K_2_0 | Organic matter | CaCo_3_ |
| --- | --- | --- | --- | --- | --- | --- | --- |
|  |  |  | Mmhos cm^-1^ | Kg ha^-1^ | Kg ha^-1^ | % | % |
| 2016 | 1 | 7.70 | 1.21 | 2.071 | 17.19 | 2.08 | 30.19 |
| 2016 | 2 | 7.74 | 1.15 | 2.019 | 17.19 | 1.98 | 30.74 |
| Mean |  | 7.72 | 1.18 | 2.045 | 17.19 | 2.03 | 30.465 |
|  |  |  |  |  |  |  |  |
| 2017 | 1 | 8.21 | 0.71 | 0.888 | 28.57 | 1.30 | 31.57 |
| 2017 | 2 | 8.20 | 0.66 | 0.853 | 28.59 | 1.35 | 28.27 |
| Mean |  | 8.205 | 0.685 | 0.8705 | 28.58 | 1.33 | 29.92 |

^a^Source: This report was provided by Directorate of Soil Water and Combating Desertification Research Institute, Ministry of Food Agriculture and Livestock, Konya, Turkey.

**Soil sample analysis**

Two sets of 20 soil sub-samples (30 cm deep) were randomly taken in a zig-zag fashion from each year’s field experiment and a composite soil sample was prepared after thoroughly mixing 20 sub-samples. Soil analysis report was provided by Directorate of Soil Water and Combating Desertification Research Institute, Ministry of Food Agriculture and Livestock, Konya, Turkey.
